# BET inhibitor suppresses migration of human hepatocellular carcinoma by inhibiting SMARCA4

**DOI:** 10.1038/s41598-021-91284-2

**Published:** 2021-06-03

**Authors:** Hae In Choi, Ga Yeong An, Mina Baek, Eunyoung Yoo, Jin Choul Chai, Young Seek Lee, Kyoung Hwa Jung, Young Gyu Chai

**Affiliations:** 1 Department of Bionanotechnology, Hanyang University, Seoul, 04673 Republic of Korea; 2 Institute of Natural Science and Technology, Hanyang University, Ansan, 15588 Republic of Korea; 3 Department of Molecular and Life Science, Hanyang University, Ansan, 15588 Republic of Korea; 4 College of Veterinary Medicine, Seoul National University, Seoul, 08826 Republic of Korea; 5 Convergence Technology Campus of Korea Polytechnic II, Incheon, 21417 Republic of Korea; 6 Department of Biopharmaceutical System, Gwangmyeong Convergence Technology Campus of Korea Polytechnic II, Gwangmyeong , 14222 Republic of Korea

**Keywords:** Cancer, Molecular biology

## Abstract

Hepatocellular carcinoma (HCC) is one of the most prevalent and poorly responsive cancers worldwide. Bromodomain and extraterminal (BET) inhibitors, such as JQ1 and OTX-015, inhibit BET protein binding to acetylated residues in histones. However, the physiological mechanisms and regulatory processes of BET inhibition in HCC remain unclear. To explore BET inhibitors’ potential role in the molecular mechanisms underlying their anticancer effects in HCC, we analyzed BET inhibitor-treated HCC cells’ gene expression profiles with RNA-seq and bioinformatics analysis. BET inhibitor treatment significantly downregulated genes related to bromodomain-containing proteins 4 (BRD4), such as ACSL5, SLC38A5, and ICAM2. Importantly, some cell migration-related genes, including AOC3, CCR6, SSTR5, and SCL7A11, were significantly downregulated. Additionally, bioinformatics analysis using Ingenuity Knowledge Base Ingenuity Pathway Analysis (IPA) revealed that SMARCA4 regulated migration response molecules. Furthermore, knockdown of SMARCA4 gene expression by siRNA treatment significantly reduced cell migration and the expression of migration-related genes. In summary, our results indicated that BET inhibitor treatment in HCC cell lines reduces cell migration through the downregulation of SMARCA4.

## Introduction

Hepatocellular carcinoma (HCC) is one of the most prevalent cancers worldwide. It is one of the leading causes of cancer-related death, with a high incidence and high mortality^1,2^. HCC initiation and progression are driven by the accumulation of several aberrations and dysregulation of the genome and epigenome^3–6^. These anomalies induce gene expression changes and give way to highly regulated gene expression, including gene networks and epigenetic modifications in essential for cell death, cell migration, and tumor growth^7–11^.

Bromodomain and extraterminal (BET) family proteins act as a transcriptional coactivator by playing a super-enhancer organization and oncogene expression regulation^12^. The BET subfamily includes bromodomain-containing proteins (BRD) 2, 3, and 4 (BRD2, BRD3, and BRD4, respectively) and bromodomain testis-associated protein (BRDT). BET proteins consist of two tandem bromodomains (BD1 and BD2), an extraterminal domain, and a C-terminal domain (CTD). Specifically, BD1 and BD2 recognize acetylation patterns along with H3 histone tails^13^. BET proteins recognize the lysine residue of H2K27Ac through the acetyl-lysine binding domain of the bromodomain and regulate gene expression^14–17^.

Aberrant BRD4 expression may promote tumorigenesis in HCC cells and tumor tissue via transcriptional activation of oncogenes^18,19^. Accumulating evidence has shown the importance of BRD4 in the dysregulated expression of oncogenes in cancer^12,20–22^. Accordingly, there has been much effort to target BRD4 using anticancer drugs, but the initial evaluation of treatment with some BET inhibitors has revealed various side effects^23^. Since the BET inhibitor JQ1 reported^24^, many other clinically efficacious BET inhibitors have been identified and published, including GSK525762, birabresib (OTX-015), and ABBV-075^25^.

The efficacy of these BET inhibitors in several different cancer types has been investigated to determine if they can be used as therapeutic agents^26,27^. Among the variety of BET inhibitors, JQ1 and OTX-015 selectively bind to the BD1 and BD2 domains of BRD2, BRD3, and BRD4. BET inhibitors such as JQ1 and OTX-015 promote effects that cause cell growth inhibition, cell cycle arrest, and apoptosis in different cancer types^27,28^. Several studies on JQ1 as a treatment for HCC cells have demonstrated its anticancer activity, mainly through the suppression of c-MYC^29,30^. OTX-015 has shown antitumor effects in several cancers, including hematologic malignancies, nuclear protein in testis (NUT) midline carcinoma (NMC), leukemia, and lymphoma^31–34^. However, the efficacy of treatment of JQ1 and OTX-015 in HCC remains mostly unexplored in gene networks, and molecular mechanisms, including therapeutic and toxic side effects and precise molecular events, are only partially understood.

In this study, we performed RNA-seq analysis for the gene expression profiling of HCC cells treated with JQ1 or OTX-015 versus that of untreated HCC cells (control HCC cells). Our results show that BET inhibitor-treated HCC cell lines and untreated control groups express cellular movement and cell migration-related genes in a differential manner, allowing a better understanding of the mode of action of BET inhibitors. The cancer stage determines the prognosis and survival rate of HCC according to tumor size and metastasis, and the survival rate is significantly lower when metastasis occurs^1,35,36^. Since migration is a crucial early stage of cancer metastasis, studies are being actively pursued to treat it by suppressing migration in other cancers^37–39^. Furthermore, we show that BET inhibition mainly inhibits HCC cell migration capability through SMARCA4. The SWI/SNF complex plays a role in chromatin remodeling^40^. Widely and well mutated in various cancers, SMARCA4 is a subunit of the SWI/SNF complex^41^. SMARCA4 has been reported to be a dominant gene involved in cancer cell proliferation, migration, invasion, etc. in some cancers, such as colorectal cancer and leukemia^42,43^. Overall, the results provide useful information on molecular mechanisms for the clinical application of the cell migration abilities of HCC.

## Results

### Characterization of BET inhibitor-treated HCC cell lines

**Figure 1:**
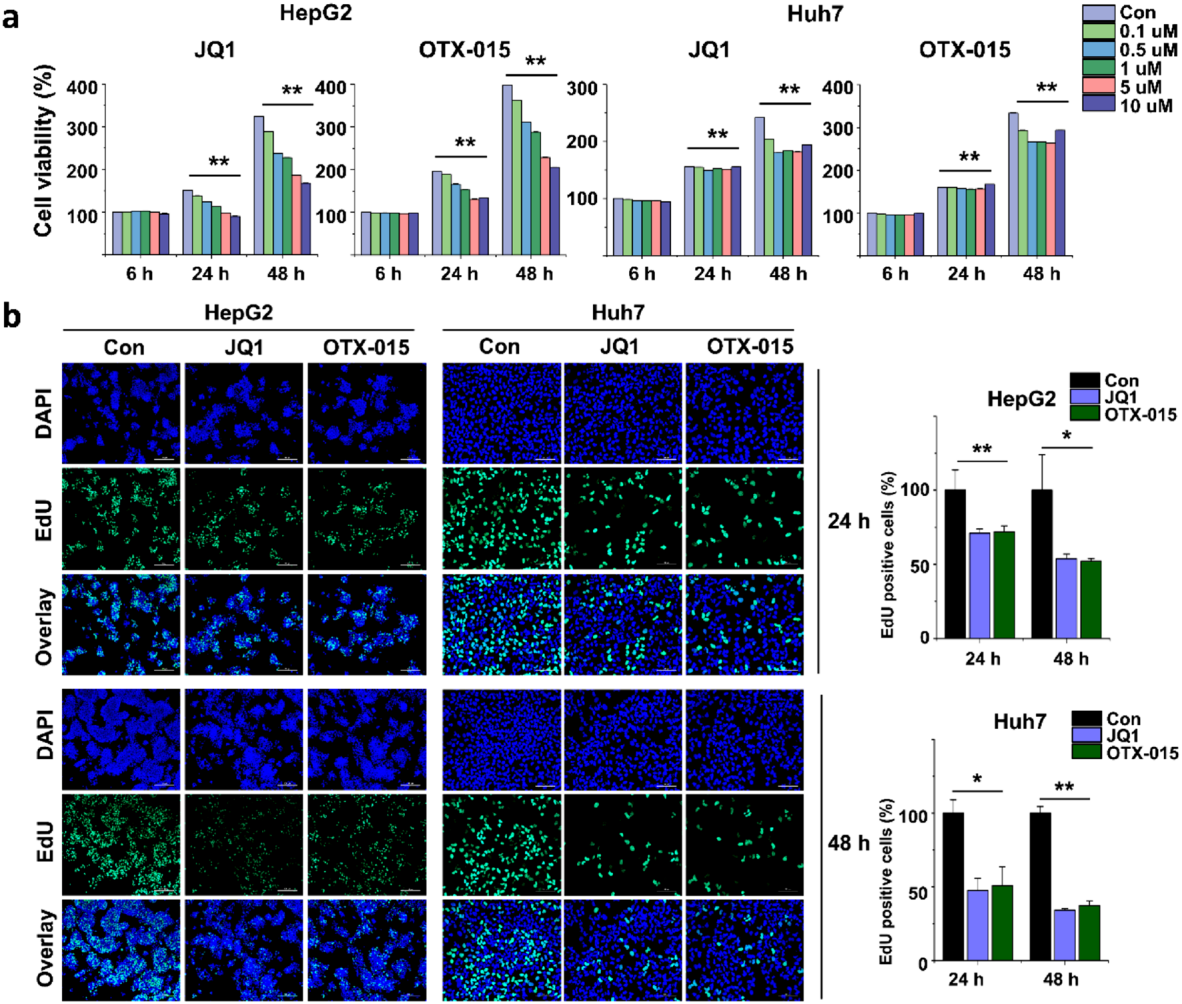
BET inhibition reduced cell proliferation of HCC. (**a**) HCC cell lines were treated with different concentrations of BET inhibitors (JQ1 or OTX-015) for different durations (6 h, 24 h, and 48 h). Cell proliferation was determined using a WST-1 assay. HCC cells treated with BET inhibitors were significantly reduced after 24 h of treatment. (**b**) Representative photo images of the EdU assay of HCC cells treated with BET inhibitors and quantification of EdU-positive cells. The data represent three biologically independent experiments. **p < 0.01.

To study the effects of BET inhibitors on HCC growth, we treated HCC cell lines (HepG2 and Huh7) with BET inhibitors (JQ1 or OTX-015) at various concentrations (0.1 µM, 0.5 µM, 1.0 µM, 5.0 µM, and 10 µM) and different durations (6 h, 24 h, and 48 h). BET inhibitor treatment resulted in significantly reduced HCC cell line proliferation in a concentration-dependent manner after 24 h of treatment (Fig. 1a). Moreover, we performed a proliferation assay of the HCC cell lines treated with BET inhibitors for 24 h and 48 h. We found that BET inhibition decreased the proportion of EdU-positive cells for 24 h and 48 h, indicating that BET inhibition reduced the proliferation of HCC cell lines. These results suggest that HCC cell line proliferation was inhibited in BET inhibitor-treated cells, as expected (Fig. 1b).

### Differentially expressed genes (DEGs) of BET inhibitor-treated HCC cells

**Figure 2:**
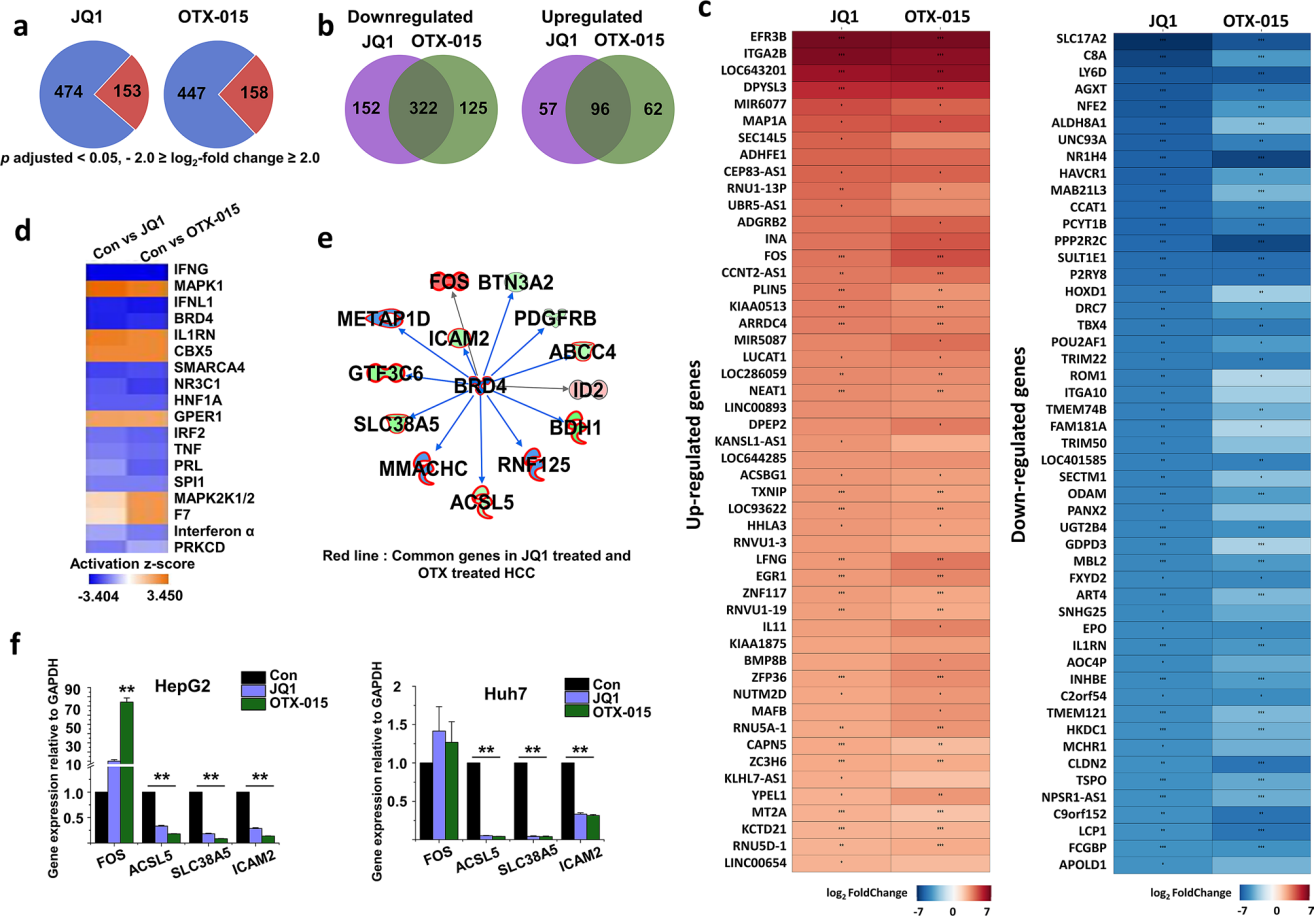
Differential gene expression in BET inhibitor-treated HCC cells and comparison of JQ1-inducible and OTX-inducible transcriptional datasets. (**a**) Pie chart displaying the number of up- and downregulated genes of BET inhibitor-treated HCC cells. Blue indicates downregulation, and red indicates upregulation. (**b**) The overlap area indicates the number of shared up- and downregulated genes in JQ1- and OTX-015-treated HepG2 cells. (**c**) A heat map representing the top 50 up- and downregulated genes in BET inhibitor-treated HepG2 cells (*p* adjusted < 0.05, log_2_-fold change ≥ 2, log_2_-fold change ≤ − 2). The color scale shown in the heat map represents the log2 fold change values. Red color indicates upregulated genes while blue color indicates downregulated genes. The heat map was created in R using the ggplot2 package version 3.3.3 (URL: https://ggplot2.tidyverse.org)^69^. The *p* value with an asterisk attached in the cell represents *p < 0.05, **p < 0.01, and ***p < 0.001. (**d**) Upstream regulator analysis of alternated gene datasets in Con vs. JQ1-treated HCC and Con vs. OTX-015-treated HCC cells using Ingenuity pathway analysis (IPA; https://www.quiagenbioinformatics.com/products/ingenuity-pathway-analysis). (**e**) The activity of highly connected negative regulators of BRD4, a member of the BET family of proteins, led to this network’s inactivation, as assessed using the IPA molecule activity predictor in BET inhibitor-treated HCC cells. The red line indicates common genes in JQ1- and OTX-015-treated HepG2 cells. (**f**) Confirmation of differentially expressed genes by qPCR in BET inhibitor-treated HCC cells compared with DMSO-treated HCC cells. The values are the mean ± S.D. of triplicate wells. **p < 0.01.

Based on the results shown in Fig. 1, we treated HepG2 cells with BET inhibitors (JQ1 or OTX-015; 5 µM) for 24 h in cDNA library preparation for RNA-seq experiment. RNA-seq transcriptional analysis was performed using three independent samples (biological replicates) of BET inhibitor treatment. We sequenced nine libraries obtained from 24 h control (3 samples), JQ1 (5 µM) (3 samples), and OTX-015 (5 µM) (3 samples) treatments. We combined the data from all experiments for each group, and the genes identified whose expression levels significantly differ. We used a 1% FDR, *P* < 0.05, fold change log_2_-fold change ≥ 2, log_2_-fold change ≤ − 2 for up- or downregulation, respectively, for defining DEGs. RNA-seq analysis revealed DEGs in BET inhibitor-treated HepG2 cells at 24 h: 627 genes in JQ1-treated HepG2 cells and 605 genes in OTX-015-treated HepG2 cells were differentially regulated. Among them, 474 and 447 genes were significantly downregulated, whereas 153 and 158 genes were statistically upregulated in JQ1- or OTX-015-treated HepG2 cells, respectively, relative to those of the control HepG2 cells after 24 h (Fig. 2a). Furthermore, to investigate the common and unique up/downregulated genes between JQ1- and OTX-015-treated HepG2 cells, we used RNA-seq data to compare the transcriptome of JQ1-treated HepG2 cells with that of OTX-015-treated HepG2 cells. JQ1-treated HepG2 cells contained 152 downregulated genes and 57 upregulated genes that were not common to OTX-015-treated HepG2 cells. In contrast, OTX-015-treated HepG2 cells had 125 downregulated genes and 62 upregulated genes that were not common to JQ1-treated HepG2 cells (Fig. 2b). However, JQ1- and OTX-015-treated HepG2 cells also had similarities in their transcriptomes. Of the downregulated genes, JQ1- and OTX-015-treated HepG2 cells shared 322 genes. Of the upregulated genes, JQ1- and OTX-015-treated HepG2 cells shared 96 genes (Fig. 2c).

To further characterize the BET inhibitor-treated HepG2 cells, we performed an upstream regulator analysis of DEGs using IPA software. The upstream regulator analysis identified 18 regulators, of which the top regulators were IFNG, MAPK1, IFNL1, and BRD4 (Fig. 2d). IPA analysis indicated that BRD4 formed a direct or indirect network with several downregulated genes commonly involved in JQ1- and OTX-015-treated HepG2 cells (Fig. 2e). The expression changes of these genes, including FOS, ACSL5, SLC38A5, and ICAM2, were validated by qPCR using GAPDH as the reference gene (Fig. 2f). To confirm BET inhibitors’ distinct effects in HepG2 cells, we incubated HepG2 cells treated with JQ1 or OTX-015, which showed that ACSL5, SLC38A5, and ICAM2 were downregulated and FOS was upregulated. More importantly, BET inhibitors also suppressed genes’ expression, including ACSL5, SLC38A5, and ICAM2, in Huh7 cells.

### Network analysis of the altered genes in BET inhibitor-treated HCC cells

**Figure 3:**
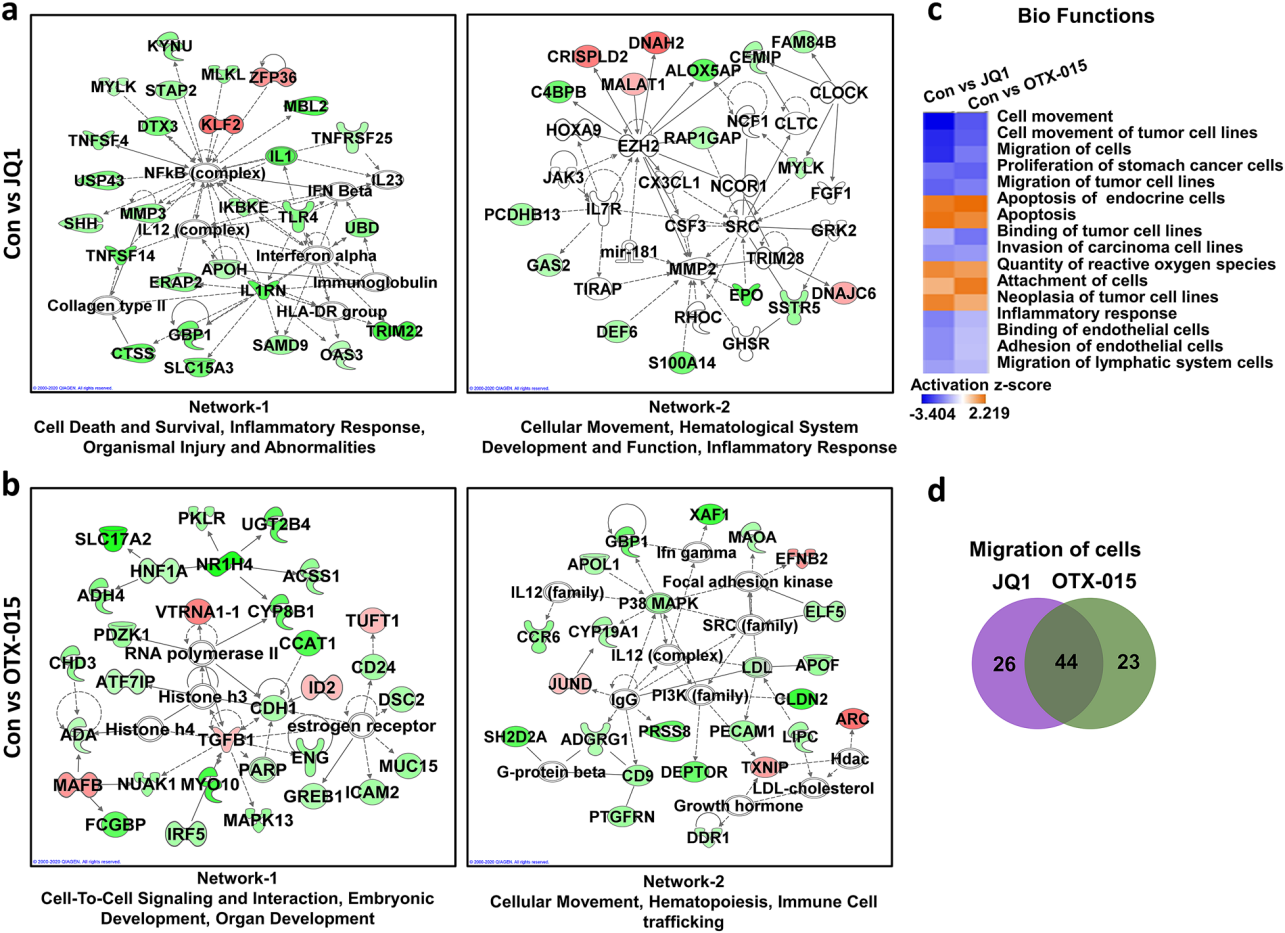
IPA-based network analysis of JQ1- or OTX-015-treated HCC cells. (**a**) Gene networks of JQ1- or (**b**) OTX-015-treated HCC cells by IPA. (**c**) Biofunctional analysis of alternated gene datasets in Con vs. JQ1-treated HCC and Con vs. OTX-015-treated HCC cells using IPA. (**d**) The area of overlap indicates the number of shared genes that related with migration of cells in JQ1 and OTX-015 treated HepG2.

Next, we identified the network of genes and related pathways representing the interacting genes in JQ1- or OTX-015-treated HepG2 cells using IPA software. Network-1 and Network-2 in JQ1- or OTX-015-treated HepG2 cells are illustrated in Figure 3a and b. Twenty-four hours of JQ1 treatment revealed genes in networks-1 known to be involved in cell death and survival, inflammatory response, organismal injury, and abnormalities (Fig. 3a). The genes in Network-2 in JQ1-treated HepG2 cells are involved in cellular movement, hematological system development and function, and inflammatory response. In OTX-015-treated HepG2 cells, the genes in Networks-1 are known to be involved in cell-to-cell signaling and interaction, embryonic development, and organ development. The genes in Networks-2 in OTX-015-treated HepG2 cells are known to be involved in cellular movement, hematopoiesis, and immune cell trafficking (Fig. 3b).

### Biofunctional analysis of the altered genes in BET inhibitor-treated HCC cells

To further characterize BET inhibitor-treated HepG2 cells, we determined the biofunctions of DEGs obtained from JQ1- or OTX-015-treated HepG2 cells (Fig. 3c). The gene functions activated by JQ1 or OTX-015 treatment were generally associated with apoptosis, reactive oxygen species, and cell adhesion. Interestingly, the gene functions that were inactivated by JQ1 or OTX-015 treatment were genes commonly associated with cell migration.

Furthermore, to investigate the common and unique migration-regulated genes between JQ1- and OTX-015-treated HepG2 cells, we used RNA-seq data to compare the transcriptome of JQ1-treated HepG2 cells with that of OTX-015-treated HepG2 cells (Fig. 3d). JQ1-treated HepG2 cells demonstrated 26 changed genes that were not common to the OTX-015-treated HepG2 cells. In contrast, OTX-015-treated HepG2 cells showed 23 altered genes that were not common to JQ1-treated HepG2 cells. Of the changed genes, JQ1- and OTX-015-treated HepG2 cells shared 44 genes.

### Downregulation of cell migration-related genes in BET inhibitor-treated HCC cells

**Figure 4:**
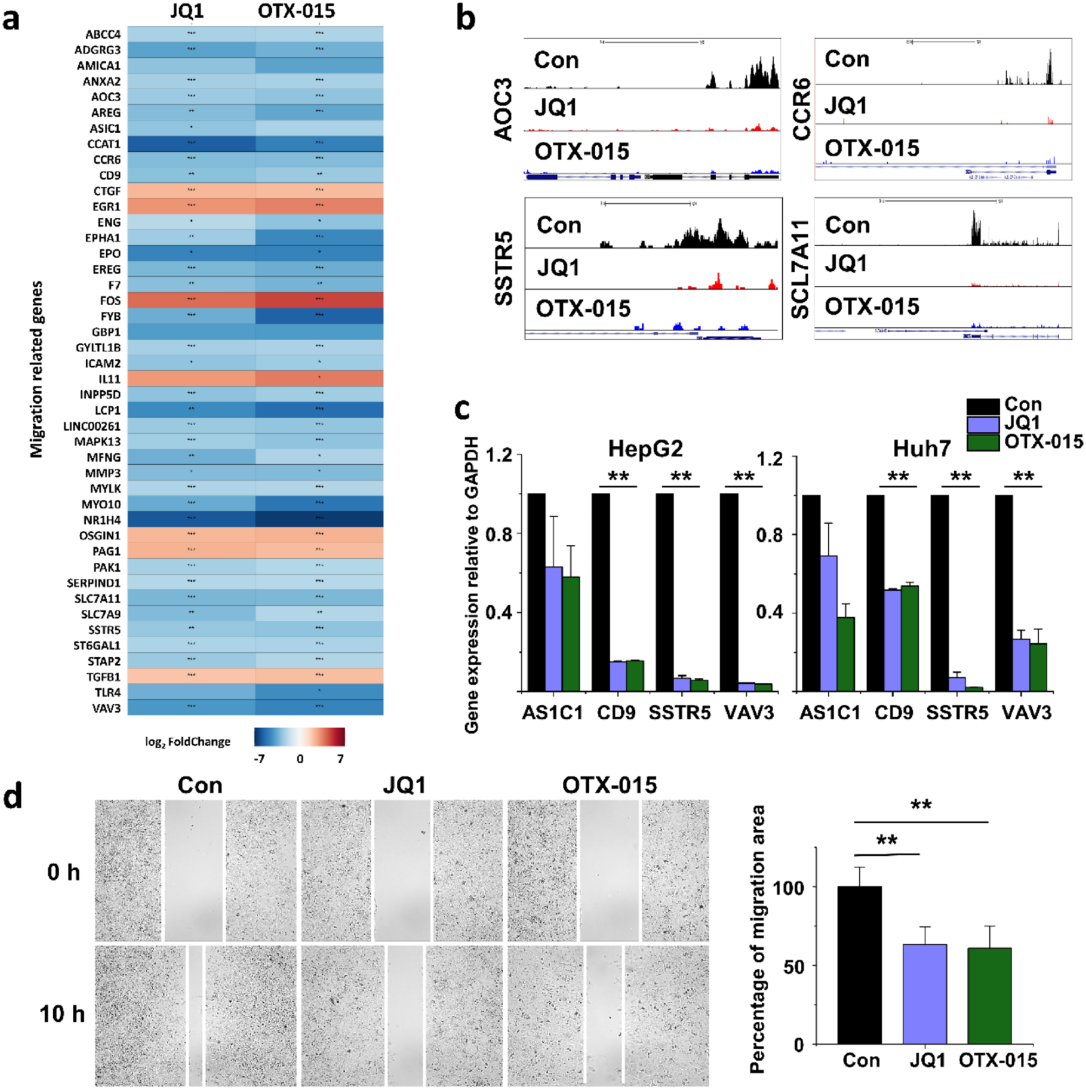
Downregulation of cell migration-related genes in BET inhibitor-treated HCC cells. (**a**) A heat map representing the migration-related genes in BET inhibitor-treated HepG2 cells compared with those in the controls. The color scale shown in the heat map represents the log2 fold change values. Red color indicates upregulated genes while blue color indicates downregulated genes. The heat map was created in R using the ggplot2 package version 3.3.3 (URL: https://ggplot2.tidyverse.org)^69^. The *p* value with an asterisk attached in the cell represents *p < 0.05, **p < 0.01, and ***p < 0.001. (**b**) The UCSC genome browser images show normalized RNA-seq read densities in control, JQ1-treated, and OTX-015-treated HCC cells. (**c**) Confirmation of differentially expressed genes using qPCR in JQ1- or OTX-015-treated HCC cells. The values are the mean ± S.D. from triplicate well measurements. **p < 0.01. The data represent three independent experiments. (**d**) The migration of HCC cells was determined using a wound-healing assay. Migrating HCC cells were determined after 10 h of JQ1 or OTX-015 treatment. The area filled with HCC cells that entered the middle blank fields was calculated. The data represent three biologically, independent experiments. **p < 0.01.

After a functional analysis of the altered genes in BET inhibitor-treated HepG2 cells, we focused on those associated with cell migration. Of these cell migration-related genes, the 44 commonly altered genes are listed in Figure 4a. The normalized RNA-seq read densities of cell migration-related genes (AOC3, CCR6, SSTR5, and SCL7A11) were decreased in BET inhibitor-treated HepG2 cells (Fig. 4b). To verify the RNA-seq results, we confirmed the expression of cell migration-related genes by qPCR. ASIC1, CD9, SSTR5, and VAV3 mRNA were downregulated in BET inhibitor-treated HepG2 and Huh7 cells (Fig. 4c).

Besides, a wound healing assay was conducted to investigate the migration effects of BET inhibitor-treated cells. The number of Huh7 cells that migrated into a wound field following BET inhibitor treatment was significantly smaller than that of control cells (Fig. 4d). These data strongly suggest that BET inhibitor treatment was associated with the cell migration response.

### Role of SMARCA4 in cell proliferation and migration

**Figure 5:**
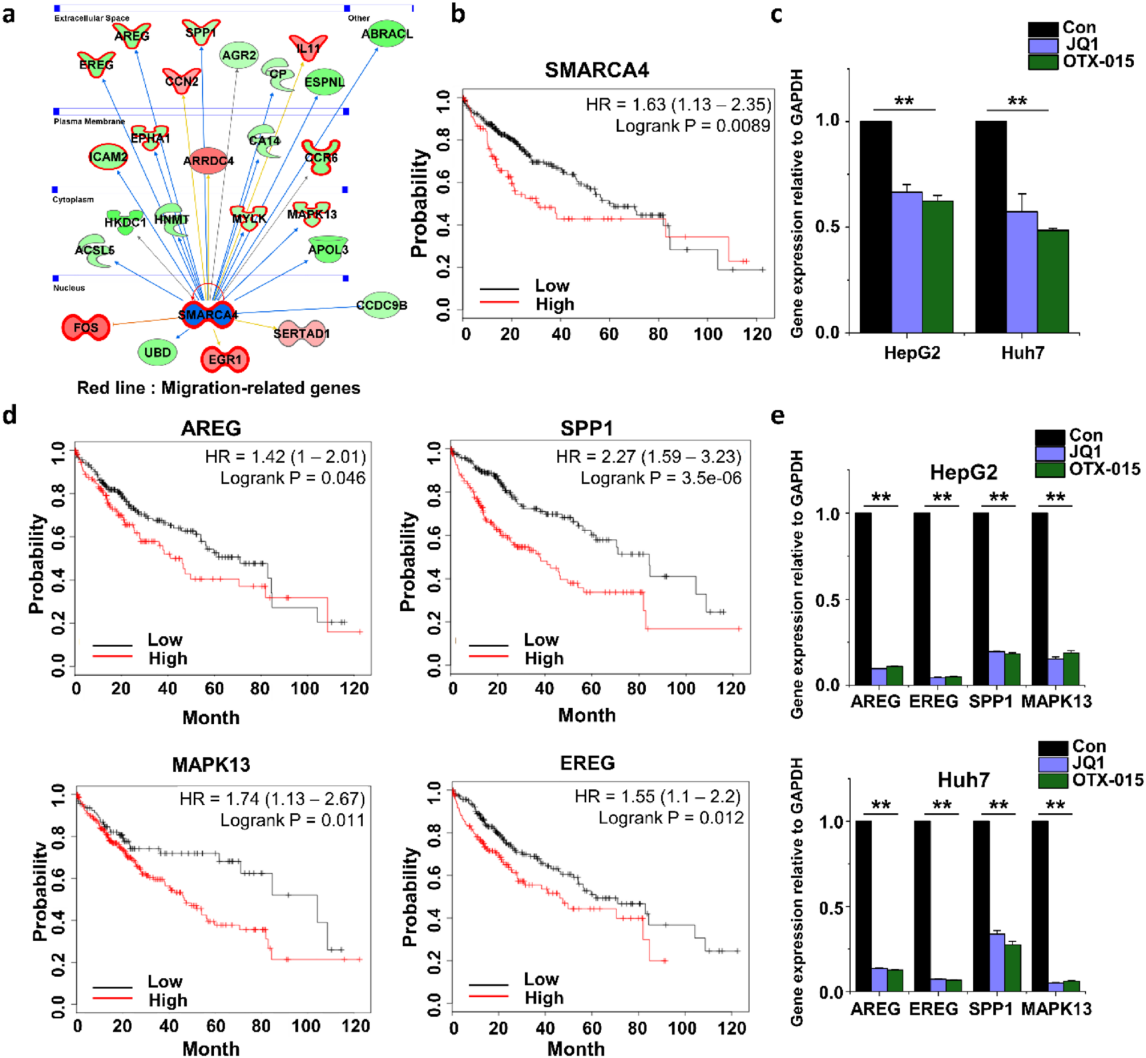
Differences in the expression of migration-related genes. (**a**) Migration response molecules were analyzed with an IPA molecule activity predictor. Shown are the migration response molecules regulated by SMARCA4. The red line indicates migration-related genes. (**b**) Kaplan–Meier plotter analysis for overall survival of HCC patients was divided into high and low SMARCA4 expression groups. The result indicated that patients with low SMARCA4 expression had a better prognosis than those with high SMARCA4 expression. (**c**) Confirmation of SMARCA4 expression levels in JQ1- or OTX-015-treated HCC cells. (**d**) Kaplan–Meier plotter analysis for overall survival of HCC patients was divided into high and low AREG, SPP1, MAPK13, and EREG expression groups. (**e**) Confirmation of the expression levels of AREG, EREG, SPP1, and MAPK13 in JQ1- or OTX-015-treated HCC cells. The values are the mean ± S.D. of triplicate well measurements. **p < 0.01.

The initial bioinformatics analysis revealed that many genes involved in cell migration were regulated by SMARCA4 (Fig. 5a). Kaplan–Meier survival curve analysis suggested that the overall survival of liver cancer patients with high SMARCA4 expression was shorter than that of patients with low SMARCA4 expression (Fig. 5b). We analyzed the expression of the SMARCA4 gene using qPCR. The SMARCA4 gene expression was significantly downregulated in BET inhibitor-treated HCC cell lines (Fig. 5c).

To further verify bioinformatics analysis results, we analyzed the Kaplan–Meier survival curves of genes (AREG, SPP1, MAPK13, and EREG) related to cell migration regulated by SMARCA4. Kaplan–Meier survival curve analysis suggested that liver cancer patients’ overall survival with high expression of these genes was shorter than that of patients with low expression (Fig. 5d). The expression of AREG, SPP1, MAPK13, and EREG genes obtained using qPCR was significantly downregulated in BET inhibitor-treated HCC cell lines (Fig. 5e). The HCC cell lines treated with both JQ1 and OTX-015 showed downregulated SMARCA4 genes and a few target genes regulated by SMARCA4. Thus, we speculated that SMARCA4 might play an important role in HCC cell migration.

**Figure 6:**
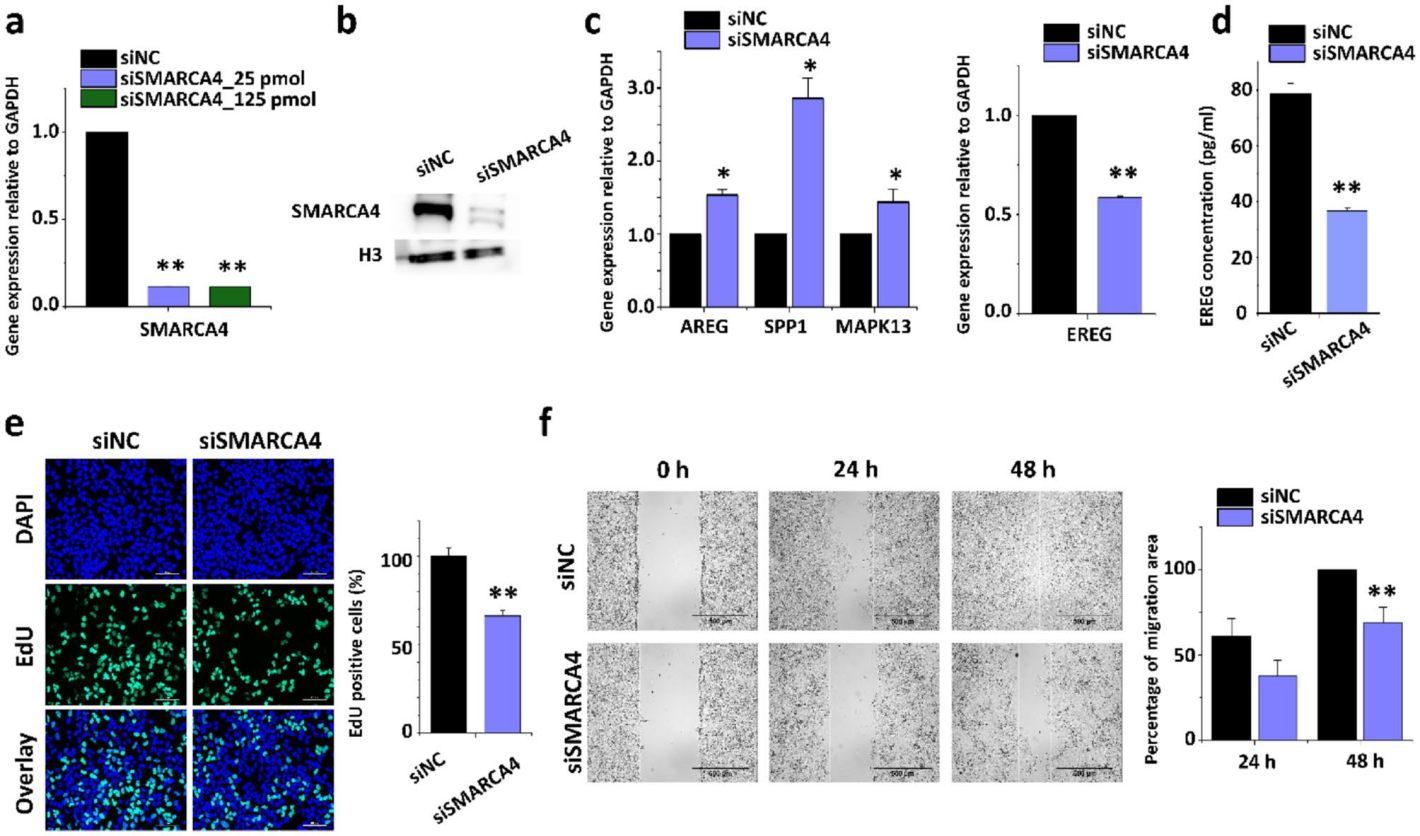
SMARCA4 regulated cell migration responses in HCC cells. (**a**) Quantitative PCRs were showing relative mRNA expression levels of SMARCA4 in scrambled siRNA control (n = 3) and SMARCA4 siRNA-treated HCC cells. Gene expression level was normalized to GAPDH. The values are the mean ± S.D. of triplicate well measurements. **p < 0.01. (**b**) Western blot was showing down-regulated SMARCA4 protein levels in siRNA-treated HCC cells. (**c**) Confirmation of the expression levels of EREG, AREG, SPP1, and MAPK13 in SMARCA4 siRNA-treated HCC cells. The values are the mean ± S.D. of triplicate wells. *p < 0.05 and **p < 0.01. (**d**) ELISA result showing the release of EREG upon repression of SMRACA4 expression. The values are means ± SD of triplicate wells. **p < 0.01. (**e**) Effect of SMARCA4 siRNA on cell proliferation by EdU assay. The data represent three biologically, independent experiments. **p < 0.01. (**f**) The migration of HCC cells was determined using a wound-healing assay. HCC cells migrated after 24 h and 48 h of SMARCA4 siRNA-treatment. The area filled with HCC cells that entered the middle blank fields was calculated. The data represent three biologically independent experiments. **p < 0.01.

Next, we investigated whether SMARCA4 depletion affects cell proliferation and cell migration. Huh7 cells were treated with different concentrations of SMARCA4 siRNA, and siSMARCA4 led to a significant lack of SMARCA4 expression relative to that in the negative control group (scrambled siRNA-siNC; Fig. 6a). These reductions were observed at the protein level (Fig. 6b). The full-length blots are presented in Supplementary Figure S2. Using qPCR analysis, we revealed that the EREG gene expression was significantly decreased in SMARCA4 depleted cells. However, some migration-related genes, including AREG, SPP1, and MAPK13, showed increased expression in SMARCA4-depleted cells (Fig. 6c). We found that SMARCA4 directly regulates EREG gene expression. Also the amount of EREG released is significantly decreased after SMARCKA4 knockdown (Fig. 6d).

Furthermore, we performed a cell proliferation assay of Huh7 cells treated with scrambled siRNA or SMARCA4 siRNA for 48 h. We found that the depletion of SMARCA4 decreased the proportion of EdU-positive cells. The decrease in SMARCA4 expression reduced the proliferation of Huh7 cells (Fig. 6e). The shortage of SMARCA4 significantly reduced the number of Huh7 cells that migrated into a wound field relative to scrambled siRNA-treated Huh7 cells (Fig. 6f and S3). These data strongly suggest that SMARCA4 is involved in the regulating cell proliferation and migration response in HCC cells.

## Discussion

HCCs are cancers that are not easy to treat due to their heterogeneity and drug resistance. Drugs, including sorafenib, a representative prescription currently used to treat HCC, have been reported to be ineffective or poorly performing in some patients^44,45^. Therefore, an epigenetic modulator was recently studied to treat cancer with kinase inhibitors^18,26,46^.

In a previous study, the BRD4 inhibitor JQ1 was reported to have an inhibitory effect on HCC cell proliferation and metastasis^18,47^. OTX-015 was confirmed to be effective for acute leukemia and NUT and was subsequently used clinically^31,33^. In this study, treatment with two representative BRD4 inhibitors, JQ1 and OTX-015, was confirmed to inhibit HCC cell lines’ proliferation. The effect of BET inhibitor OTX-015 had not been known for HCC cells. Here, we focused on common DEGs that were altered by JQ1 or OTX-015 treatment in HepG2 cells. Approximately 60–70% of the DEGs that were altered when treated with each BET inhibitor in HepG2 cells demonstrated the same pattern. It was found that each inhibitor had a reasonably similar effect. Additionally, IPA-based analysis confirmed that the typical DEGs mode after treatment with each inhibitor was related to a decrease in BRD4.

The cellular movement was critical in the IPA network analysis results for genes with decreased expression among the DEGs obtained from HepG2 cells treated with JQ1 or OTX-015. The inhibition of cell migration by JQ1 in salivary adenoid cystic carcinoma (SACC), prostate cancer, etc. through the downregulation of BRD4 had been confirmed in previous studies^48,49^. However, few studies have been conducted on cell migration by JQ1 or OTX-015 in HCC cells. Through IPA analysis, we demonstrated that genes reduced by JQ1 or OTX-015 reduce cell movement, mainly biofunctions corresponding to the migration and movement of tumor cell lines. Additionally, among DEGs, genes related to cell migration accounted for approximately 11%, and about 50% of the genes showed the same results for both drugs. These results suggest that the anticancer effect of the BET inhibitor treatment of HCC cells is due to cell migration inhibition. In addition, since DEGs corresponding to more than half showed the same pattern in JQ1-treated Huh7 cells (Fig. S1), the result suggests that it is not specific to HepG2 cells but can be interpreted as a result of HCC cells.

In HCC, the treatment method depends on the size and metastasis of the tumor. Available treatments for HCC metastasis are limited^1^. In this study, it was confirmed that cell migration, which is very important for metastasis, can be suppressed through inhibition of BRD4. Cell mobility decreased after treatment with JQ1 or OTX-015. This result is consistent with previous studies on BRD4 inhibition in HCC cells^50^.

This study studied the SMARCA4 gene to confirm that cell migration is inhibited when HCC cells are treated with JQ1 or OTX-015. SMARCA4, known as Brahma-related gene-1 (BRG1), is a component of the SWI/SWF complex (a large ATP-dependent chromatin remodeling complex) along with Brahma (BRM) and is mutated in several cancers^51^. Additionally, it is known that the SWI/SNF complex cause chromatin remodeling and affects cell differentiation and cell proliferation^51,52^. In fact, in cancers such as prostate cancer, colon cancer, and lung cancer, SMARCA4 is an epigenetic regulator and has been reported to promote metastasis through cancer migration and invasion^53–55^. It has been reported that the SMARCA4 gene is highly expressed in HCC and increases cell proliferation^52^. Our results also showed that SMARCA4 regulates a significant number of genes involved in cell migration. When the SMARCA4 gene was knocked down with siRNA, cell proliferation and mobility were reduced.

Furthermore, as a result of knocking down the SMARCA4 gene to confirm its relationship to HCC cell migration, the expression of the target gene EREG gene was reduced. EREG is a gene that encodes epiregulin, an epidermal growth factor (EGF) family member that binds to the EGF receptor (ErbB) member of the receptor tyrosine kinase family. Epiregulin’s role in tumors is to activate the Erk and PI3K kinase/Akt signaling pathways by binding to the EGF receptor. It generates proliferation, invasion, metastasis, angiogenesis, and resistance to apoptosis^56^. EREG is expressed at shallow levels in healthy cells, but it is also increased in various cancers^57^. In fact, in colorectal cancer (CRC), it has been reported that activation of the EGFR pathway through EREG demethylation may be a mechanism of several types of malignancies^58^. Especially, in salivary adenoid cystic carcinoma (SACC), ERE-induced EGFR activation has been reported as a significant cause of metastasis^59^. In HCC, an increase in epiregulin expression has been shown to act as a compensation mechanism for N-RAS’s inhibitory effect. Therefore, when N-RAS and epiregulin are simultaneously inhibited, HCC cells’ growth can be effectively suppressed^60^.

**Figure 7:**
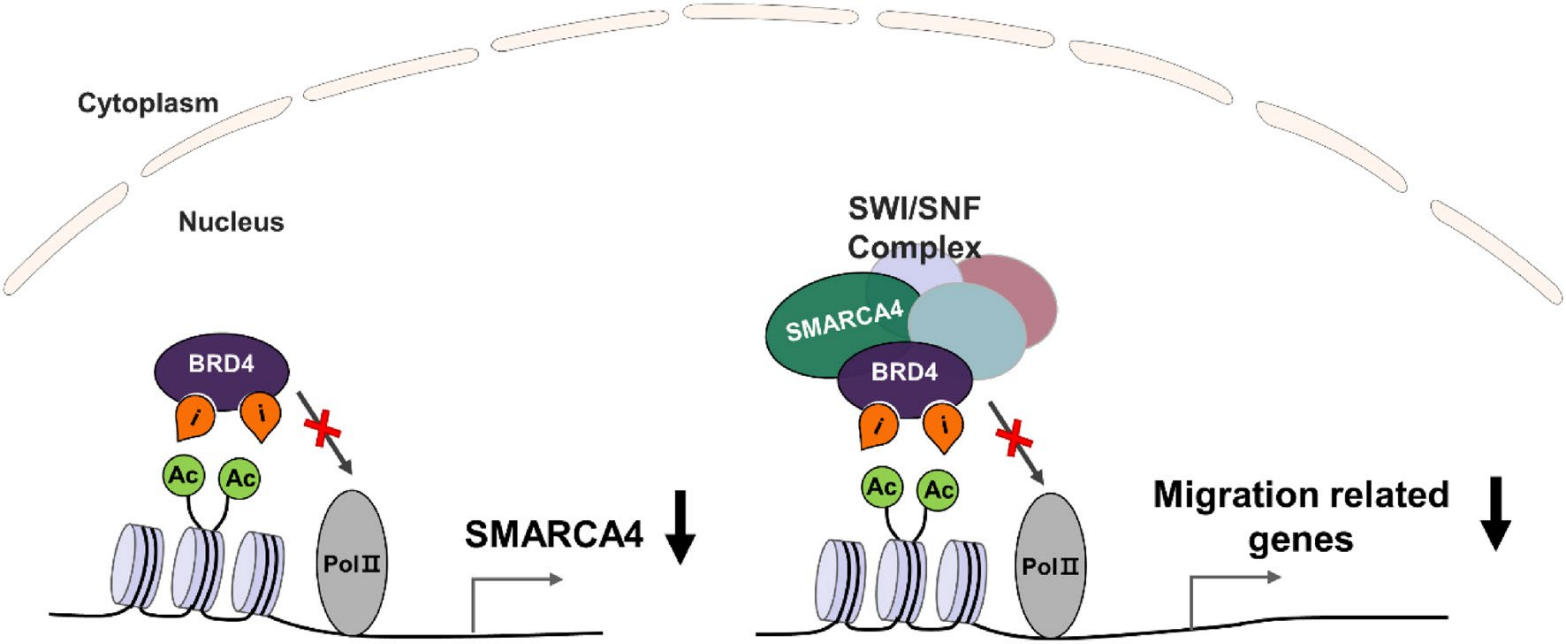
Schematic representation of SMARCA4 regulating cell migration by BET inhibitors. BRD4 and SMARCA4 have bromodomain to recognize histone acetylation and initiate transcription. SMARCA4 regulates transcription by binding to the acetyl residues of histone H3 and involves chromatin remodeling for gene expression by binding to BRD4 as an SWI/SNF complex.

Collectively, the RNA-seq data analysis results and the SMARCA4 knockdown experiments can expect two possibilities in the regulation of migration by SMARCA4 in HCC cells. The first is when a BET inhibitor suppresses the expression of SMARCA4, an upstream regulator of migration-related genes, and the second is when the recruitment of the chromatin remodeler SMARCA4 for the expression of the migration-related genes such as EREG is inhibited by inhibition of BET protein. Our models of SMARCA4 working mechanism are described in Figure 7.

The BRD4 active site is composed of two domains, BD1 and BD2. BET inhibitors such as JQ1 or OTX-015 mainly target the BD1 domain. These BET inhibitors are severely toxic and have limited clinical use^61^. Recently developed selective BD1 or BD2 inhibitors have been shown to be effective in cancer treatment or infection^62,63^. We are currently studying the pathway of genes regulated by BD2 inhibitors (such as ABBV-744) in HCC cells.

## Conclusions

In this study, we performed a transcriptome analysis of BET inhibitor-treated HCC cells. We found that BET inhibitors JQ1 and OTX-015 have a significant cancer inhibitory effect on cell proliferation and cell migration in HCC. We confirmed that BET inhibitors attenuate BRD4-related and cell migration-related genes. Primarily, cell migration-related genes regulated by SMARCA4 were reduced in BET inhibitor-treated HCC cells. These findings suggest that BET inhibitors modulate the cell migration effects of HCC and selectively inhibit the expression of cell migration-related genes through SMARCA4. This result indicates that JQ1 or OTX-015 can be used as a drug to improve HCC treatment.

## Materials and methods

### Cell culture and BET inhibitor treatment

HCC cell lines HepG2 and Huh7 were purchased from Korean Cell Line Bank (Seoul, Korea). Human hepatocellular carcinoma cell line HepG2 and Huh7 cells were cultured in MEM and RPMI-1640, respectively. And all media contained with 10% heat-inactivated FBS, penicillin and streptomycin (Thermo Fisher Scientific, Waltham, MA, USA) to maintain. The cells were cultured at 37 °C in a humidified atmosphere with 5% CO2. BET inhibitor, JQ1 and OTX-015 were purchased from Tocris Bioscience (Minneapolis, MN, USA). JQ1 and OTX-015 were dissolved in dimethyl sulfoxide (DMSO) at a stock concentration of 10 mM and stored at -20 °C. The cells were treated with various concentrations of JQ1 or OTX-015 for different lengths of time.

### Cell proliferation assay

According to the manufacturer’s instructions, the cell proliferation assay was performed using a premixed water-soluble tetrazolium salt (WST-1) cell viability test (Takara, Shiga, Japan). The cells were seeded at a density of 1 × 10^4^ cells per well and treated with JQ1 or OTX-015 for different durations (0, 12, and 48 h). WST-1 was added to each well. And the absorbance of the microplate at 450 nm was measured after an additional 4 h incubation. The data represent three independent experiments (n = 3).

Ethynyldeoxyuridine (EdU) analysis was performed using a Click-iT EdU Cell Proliferation kit (Invitrogen, CA, USA) following the manufacturer’s instructions. After that, the cells were washed with phosphate-buffered saline, mounted with a 4′,6-diamidino-2-phenylindole (DAPI)-containing mounting solution (Vectashield, Vector Laboratories, Burlingame, CA, USA), and imaged by microscopy (Nikon Eclipse 80i, Tokyo, Japan). The percentage of EdU-positive cells was examined in HCC cell lines treated with JQ1 or OTX-015 using ImageJ (Bethesda, MD, USA) software. The data represent three independent experiments (n = 3).

### Transcriptome analysis using RNA-seq

RNA sequencing (RNA-seq) was performed as previously described^64^. Total RNA was extracted from HCC cells using RNAiso Plus (Takara, Shiga, Japan) and a Qiagen RNeasy Mini kit (Qiagen, Hilden, Germany). RiboMinus Eukaryote kit (Invitrogen, Carlsbad, CA, USA) was used for Ribosomal RNA (rRNA) depletion. An RNA library was created by a NEBNext Ultra directional RNA library preparation kit from Illumina (New England BioLabs, Ipswich, MA, USA). RNA library sequencing was performed on the Illumina HiSeq2500 platform (Macrogen, Seoul, Korea). Transcriptome sequencing was performed on independent RNA samples form DMSO-treated (3 samples), JQ1-treated (3 samples), or OTX-015-treated (3 samples) HepG2 cells in biological triplicate. FASTQ files from RNA-seq were clipped and trimmed of adapters, and low-quality reads were removed using Trimmomatic^65^. These FASTQ files were aligned using STAR (version 2.7.1) aligner software with a UCSC hg38 reference^66^. Differentially expressed genes (DEGs) was analyzed using DESeq2 with the default parameters^67^. DEGs identified by RNA-seq that had an absolute log_2_-fold change larger than 2 or smaller than -2 (log_2_-fold change ≥ 2 and log_2_-fold change ≤ − 2, *p* adjusted < 0.05) were selected as DEGs in JQ1- or OTX-015-treated HepG2 cells. Heat maps were visualized in R software (3.6.2)^68^ using the ggplot2 package (3.3.3)^69^. The acquired data were deposited in the Gene Expression Omnibus database. The dataset accession number GSE158552.

### Graphical representation of the networks and pathways

The RNA-seq dataset were analyzed through the use Ingenuity Pathway Analysis (IPA; QIAGEN lnc., https://www.quiagenbioinformatics.com/products/ingenuity-pathway-analysis; Ingenuity Systems, Mountain View, CA) to analyze the networks and pathways^70^. RNA-seq data were cut off at the fold-change (log_2_-fold change ≥ 2 and log_2_-fold change ≤ -2, *p* value < 0.05) in JQ1- or OTX-015-treated HepgG2 cells. The IPA software presented a functional analysis that showed genes involved in upstream regulators, biological functions/disease, and network analysis.

### Gene expression analysis using quantitative PCR (qPCR)

Gene expression analysis was performed as previously described^64^. Total RNA was extracted from HepG2 or Huh7 cells using RNAiso Plus (Takara, Shiga, Japan) according to the manufacturer’s instructions. cDNA was synthesized by PrimeScript reverse transcriptase (Takara, Shiga, Japan) and amplified using gene-specific primers (Table 1). The primers were designed by Primer Bank (https://pga.mgh.harvard.edu/primerbank/). qPCR was performed with TBGreen Premix Ex Taq II (Takara, Shiga, Japan). We used Glyceraldehyde-3-phosphate dehydrogenase (GAPDH) as an internal control. The data represent three independent experiments (n = 3). After performing qPCR, the results were analyzed using the critical threshold (△C_T_) and the comparative critical threshold (△△C_T_) methods in ABI 7500 (Applied Biosystems, Foster City, CA, USA) software with the NormFinder and geNorm PLUS algorithms.

**Table 1:** Primer sequences used for qPCR.

qPCR	Forward (5′-3′)	Reverse (5′-3′)
FOS	CGGGCTTCAACGCAGACTA	GGTCCGTGCAGAAGTCCTG
ACSL5	CTCAACCCGTCTTACCTCTTCT	GCAGCAACTTGTTAGGTCATTG
SLC38A5	GCTACAGGCAAGAACGTGAGG	ATTCCAAACGATGTCTTCCCC
ICAM2	CGGATGAGAAGGTATTCGAGGT	CACCCACTTCAGGCTGGTTAC
ASIC1	ATGGAAAGTGCTACACGTTCAA	GTTCATCCTGACTATGGTTCTGC
CD9	AGCCATCCACTATGCGTTGA	ATGGCATCAGGACAGGACTTC
SSTR5	TGTTTGCGGGATGTTGGCT	CTGTTGGCGTAGGAGAGGA
VAV3	AGAGAAACGGACCAATGGACT	GGTGGTGTTCCAGAATAGTTCC
SMARCA4	AATGCCAAGCAAGATGTCGAT	GTTTGAGGACACCATTGACCATA
AREG	GTGGTGCTGTCGCTCTTGATA	CCCCAGAAAATGGTTCACGCT
EREG	GTGATTCCATCATGTATCCCAGG	GCCATTCATGTCAGAGCTACACT
SPP1	ACTCGAACGACTCTGATGATGT	GTCAGGTCTGCGAAACTTCTTA
MAPK13	TGAGCCGACCCTTTCAGTC	AGCCCAATGACGTTCTCATGC

### In vitro wound-healing assay

HCC cells were seeded into each Culture-Insert (Ibidi, Martinsried, Germany) and incubated for 24 h. The cells were treated with JQ1 or OTX-015 for 24 h or transfected with scrambled siRNA or SMARCA4 siRNA for 48 h. After incubation, the inserts were removed to create a “wound field.” The cells were washed once and incubated with growth media for 24 h or 48 h. The cells were then visualized using a JuLi BR real-time cell history recorder (NanoEnTek, Seoul, Korea).

### Kaplan–Meier plotter analysis for overall survival of HCC

We used Kaplan–Meier plotter (KM plotter; http://kmplot.com/analysis/), an online biomarker analysis tool, that evaluates the prognostic value of biomarkers in various cancers. In liver cancer data, 364 cases were analyzed, and the false discovery rate (FDR) cutoff was set to 1%^71^. Briefly, to obtain KM survival plots of the EREG, AREG, SPP1, MAPK13 genes in HCC, those genes were entered into the database.

### Knockdown of SMARCA4 gene expression using siRNA treatment

Knockdown (KD) of gene expression was performed using small interfering RNA (siRNA). After seeding the cells, transfection was performed using Lipofectamine RNAiMax (Invitrogen, CA, USA) transfection agent according to the manufacturer’s instructions with siRNA constructs and scrambled siRNAs. SMARCA4 siRNA (ID numbers 4677; 5′-GCAUUUCAAGGAAUAUCACtt-3′) and Silencer Negative Control siRNA (AM4611) were purchased from Thermo Fisher Scientific. SMARCA4 siRNA and scrambled siRNA were used at a 10 nM concentration for 48 h in a growth medium.

### Western blotting assay

According to the manufacturer’s instructions, nuclear or cytoplasmic proteins from the cells were isolated using a NE-PER Nuclear Cytoplasmic Extraction Reagent Kit (Thermo Fisher Scientific, Waltham, USA). Nuclear protein was separated by sodium dodecyl sulfate (SDS) polyacrylamide gel electrophoresis (SDS-PAGE) and transferred to polyvinylidene difluoride membranes (Schleicher & Schuell Bioscience, Inc., Keene, NH, USA). Western blot analysis was performed using anti-SMARCA4 (Abcam, Cambridge, UK; ab110641) and anti-histone H3 (Abcam, Cambridge, UK; ab1791) antibodies. Histone H3 protein was used as an internal control.

### Chemokine measurements with enzyme-linked immunosorbent assay (ELISA)

Huh7 cells were transfected with siRNA for 48 h. After transfected, cell culture supernatants were concentrated 20-fold using Pierce Protein Concentrator (Thermo Fisher Scientific, Waltham, MA, USA). According to the manufacturer’s instructions, the concentration of the EREG in concentrated supernatants was determined using human EREG ELISA kits (Abcam, Cambridge, UK; ab277077). The data represent three independent experiments (n = 3).

### Statistical analysis

Data are presented as the mean ± standard deviation (SD) of the mean. All statistical analyses were performed using IBM SPSS Statistics 26.0 program (IBM corp., Armonk, NY). We used one-way analysis of variance followed by Tukey’s honestly significant difference post hoc test.* p* values < 0.05 were considered significant.

## Supplementary Information

Supplementary Information.

## Abbreviations

HCC: Hepatocellular carcinoma
BRD4: Bromodomain-containing proteins 4
SMARCA4: SWI/SNF related, matrix associated, actin-dependent regulator of chromatin, subfamily a, member 4
DEG: Differentially expressed genes
qPCR: Quantitative reverse transcription-polymerase chain reaction
RNA-seq: RNA-sequencing
