# Supplementary material for: BET inhibitor suppresses migration of human hepatocellular carcinoma by inhibiting SMARCA4
Source: Sci Rep. 2021 Jun 3;11:11799. doi: 10.1038/s41598-021-91284-2 (PMC8175750; doi:10.1038/s41598-021-91284-2)
Supplement: Supplementary file 1 — Supplementary Information. [file 41598_2021_91284_MOESM1_ESM.docx]

**BET inhibitor suppresses migration of human hepatocellular carcinoma by inhibiting SMARCA4**

Hae In Choi^1‡^, Ga Yeong An^1‡^, Mina Baek^2,3^, Eun Yeong Yoo^1^, Jin Choul Chai^4^, Young Seek Lee^4**^, Kyoung Hwa Jung^5**^, Young Gyu Chai^1,3*^

^1^ Department of Bionanotechnology, Hanyang University, Seoul, 04673, Republic of Korea.

^2^ Institute of Natural Science and Technology, Hanyang University, Ansan, 15588, Republic of Korea.

^3^ Department of Molecular & Life Science, Hanyang University, Ansan, 15588, Republic of Korea

^4^ College of Veterinary Medicine, Seoul National University, Seoul, 08826, Republic of KOREA

^5^Convergence Technology Campus of Korea Polytechnic II, Incheon, 21417, Republic of Korea.

^‡^ These authors have contributed equally to this work

* Corresponding authors

**Supplementary figures**

**Figure S1.**

**
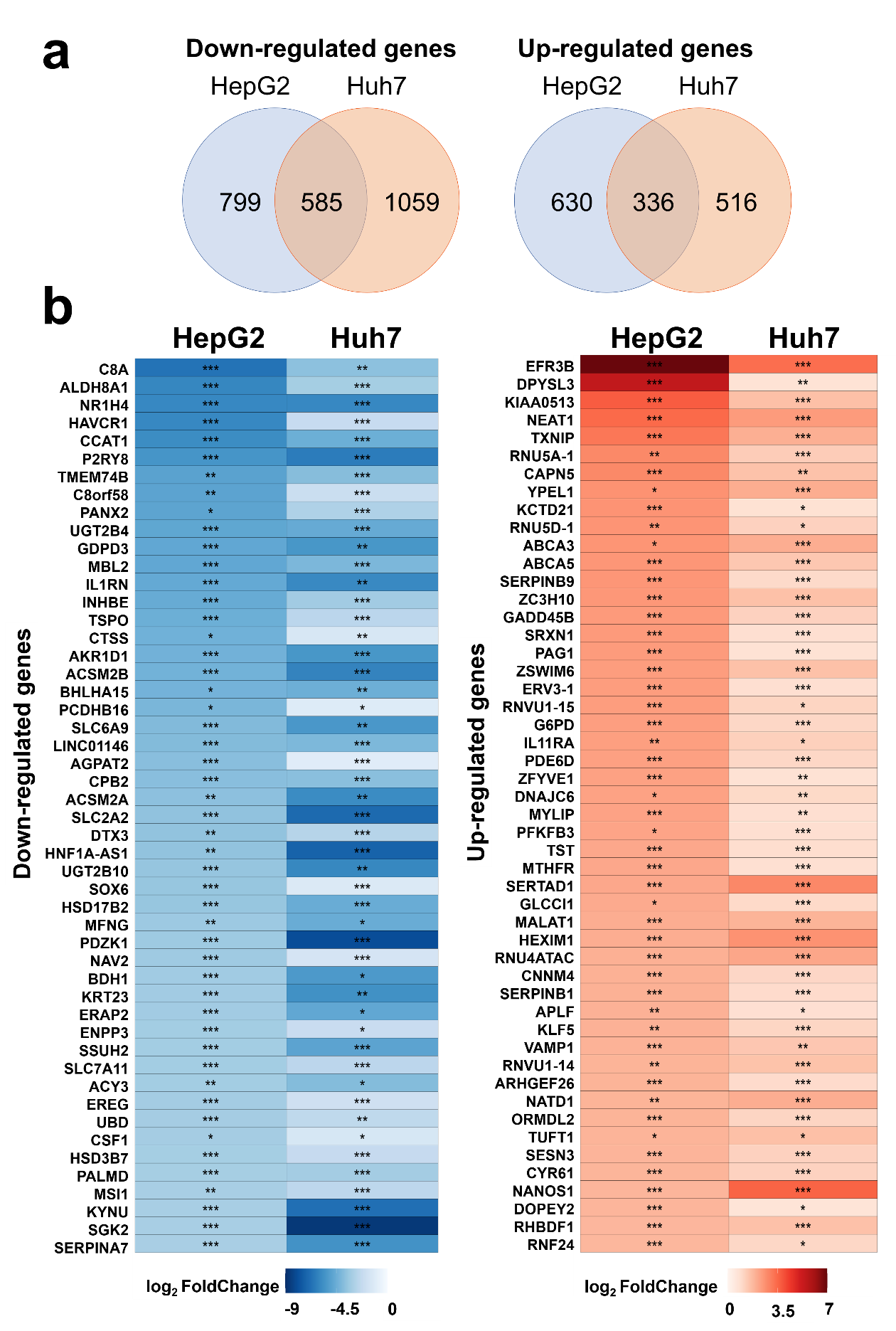
**

**Figure S1. Comparison of differential gene expression between JQ1-treated HepG2 cells and JQ1-treated Huh7 cells**

(a) The overlap area indicates the number of shared up- and downregulated genes in JQ1-treated HepG2 and JQ1-treated Huh7 cells (*p* adjusted < 0.05, log_2_-fold change ≥ 0.7, log_2_-fold change ≤ -0.7). (b) A heat map representing the top 50 up- and downregulated genes in JQ1-treated HepG2 cells, and JQ1-treated Huh7 cells. The color scale shown in the heat map represents the log2 fold change values. Red column indicate upregulated genes while blue column indicate downregulated genes. The heat map was created in R using the ggplot2 package version 3.3.3 (URL: https://ggplot2.tidyverse.org) ^69^. The *p*-value with an asterisk attached in the cell represents * *p* < 0.05, ** *p* < 0.01, and *** *p* < 0.001.

**Figure S2.**

**
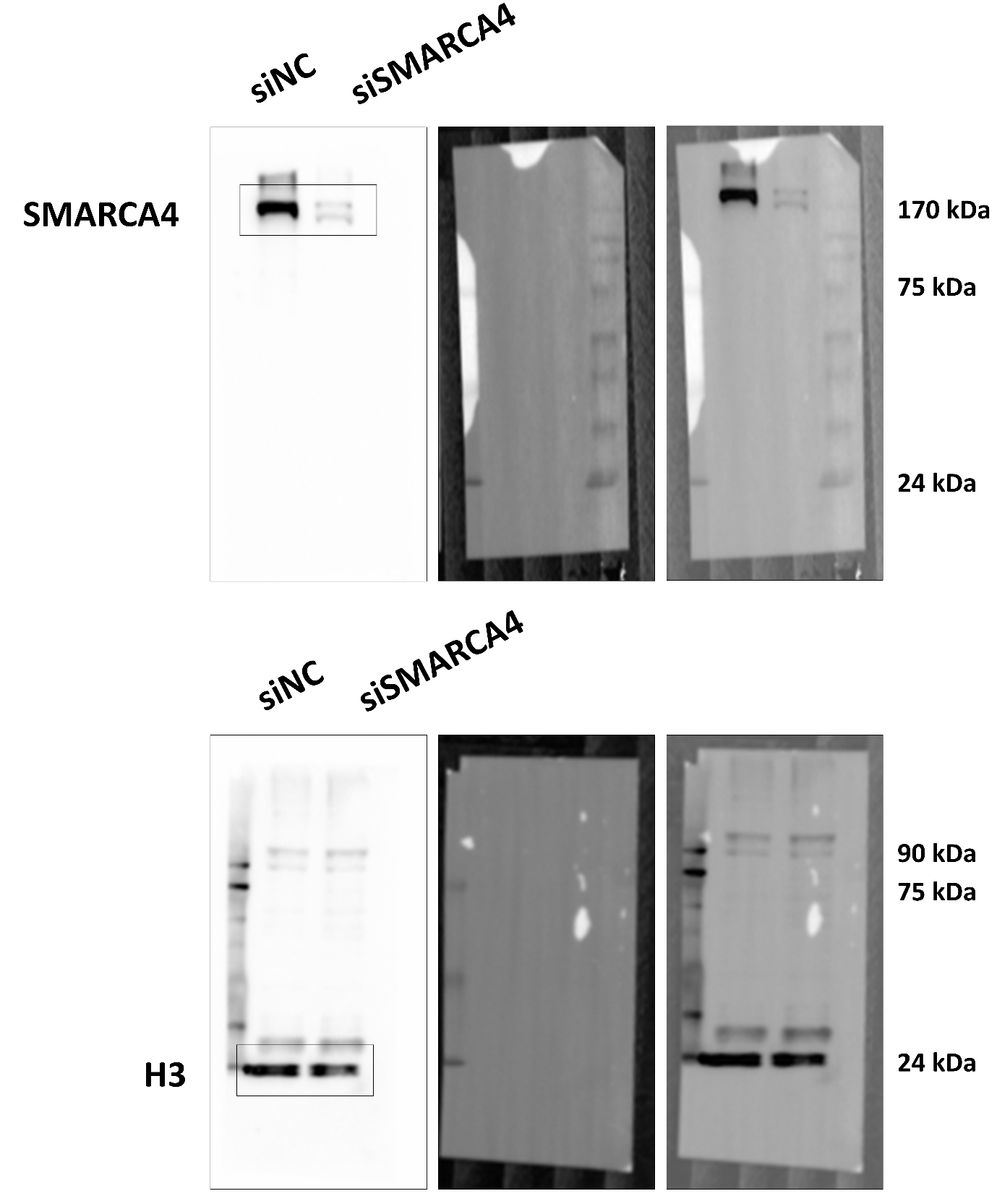
**

**Figure S2. Full-length blots image of SMARCA4**

Figure S2 is a full-length blots in Figure 6b. The left image shows the detected band, the middle image shows the membrane, and the right image has merged both images, respectively.

**Figure S3.**

**
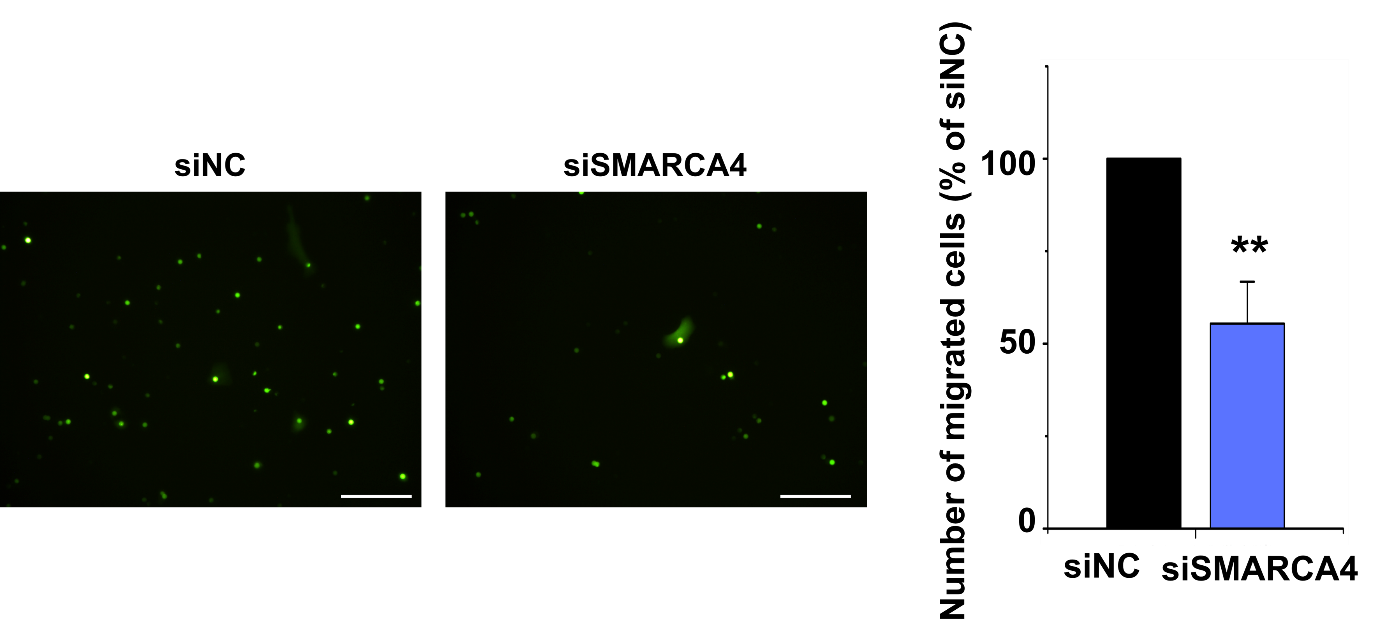
**

**Figure S3. Cell migration is suppressed in SMARCA4 inhibited HCC cells**

Cell migration was measured using a Transwell assay in HCC cells after SMARCA4 siRNA transfected for 48 hours. Cells were stained with calcein AM. Scale bar = 100 μm. The data represent three biologically independent experiments. **, *p* < 0.01.

Supplementary Table 1: Top 100 significant downregulated genes in JQ1 treated HepG2 cells

| **Gene Accession_ID** | **Gene Symbol** | **log_2_FoldChange** | **padj** |
| --- | --- | --- | --- |
| NM_005835 | SLC17A2 | -7.2 | 0.02726 |
| NM_000562 | C8A | -6.7 | 0.000668 |
| NM_003695 | LY6D | -6.2 | 0.037185 |
| NM_000030 | AGXT | -6.2 | 4.95E-07 |
| NM_001136023 | NFE2 | -6.2 | 0.016078 |
| NM_022568 | ALDH8A1 | -6.0 | 2.56E-32 |
| NM_001143947 | UNC93A | -6.0 | 0.094484 |
| NM_001206978 | NR1H4 | -6.0 | 0.017663 |
| NM_001308156 | HAVCR1 | -5.9 | 0.017427 |
| NM_152367 | MAB21L3 | -5.8 | 0.036384 |
| NR_108049 | CCAT1 | -5.8 | 0.00399 |
| NM_001163264 | PCYT1B | -5.8 | 0.067454 |
| NM_181876 | PPP2R2C | -5.7 | 0.065745 |
| NM_005420 | SULT1E1 | -5.6 | 1.43E-26 |
| NM_178129 | P2RY8 | -5.5 | 0.054011 |
| NM_024501 | HOXD1 | -5.2 | 0.024816 |
| NM_001289162 | DRC7 | -5.2 | 0.001946 |
| NM_018488 | TBX4 | -5.1 | 0.033292 |
| NM_006235 | POU2AF1 | -5.1 | 0.019629 |
| NM_001199573 | TRIM22 | -5.1 | 0.037746 |
| NM_000327 | ROM1 | -5.0 | 0.068654 |
| NM_001303041 | ITGA10 | -5.0 | 0.030095 |
| NM_018354 | TMEM74B | -5.0 | 7.61E-40 |
| NM_138344 | FAM181A | -4.9 | 0.007823 |
| NM_153338 | GGT6 | -4.9 | 0.016882 |
| NM_178125 | TRIM50 | -4.9 | 0.098021 |
| NR_125365 | LOC401585 | -4.9 | 0.034841 |
| NM_173686 | C8orf58 | -4.9 | 0.00346 |
| NM_003004 | SECTM1 | -4.9 | 0.079288 |
| NM_017855 | ODAM | -4.9 | 0.053577 |
| NM_001160300 | PANX2 | -4.9 | 0.063906 |
| NM_001297615 | UGT2B4 | -4.8 | 6.25E-05 |
| NM_024307 | GDPD3 | -4.8 | 1.28E-05 |
| NM_000242 | MBL2 | -4.8 | 0.037686 |
| NM_021603 | FXYD2 | -4.7 | 5.43E-06 |
| NM_021071 | ART4 | -4.7 | 3.00E-15 |
| NM_006404 | PROCR | -4.7 | 0.000257 |
| NR_132278 | SNHG25 | -4.7 | 0.083014 |
| NM_000799 | EPO | -4.7 | 0.008478 |
| NM_000577 | IL1RN | -4.7 | 2.71E-08 |
| NR_002773 | AOC4P | -4.7 | 6.82E-05 |
| NM_031479 | INHBE | -4.6 | 0.027122 |
| NM_024861 | C2orf54 | -4.6 | 0.000989 |
| NM_001331238 | TMEM121 | -4.6 | 0.116362 |
| NM_025130 | HKDC1 | -4.5 | 0.021984 |
| NM_080833 | RBBP8NL | -4.5 | 0.065769 |
| NM_023038 | ADAM19 | -4.5 | 6.36E-05 |
| NM_005297 | MCHR1 | -4.5 | 0.049734 |
| NM_020384 | CLDN2 | -4.5 | 0.004793 |
| NM_000714 | TSPO | -4.5 | 0.034746 |
| NM_207283 | NPSR1-AS1 | -4.4 | 0.002264 |
| NM_001012993 | C9orf152 | -4.4 | 0.00577 |
| NM_002298 | LCP1 | -4.4 | 4.50E-15 |
| NM_003890 | FCGBP | -4.4 | 0.00236 |
| NM_030817 | APOLD1 | -4.4 | 2.65E-09 |
| NM_001629 | ALOX5AP | -4.4 | 0.000172 |
| NM_004079 | CTSS | -4.4 | 0.001403 |
| NM_001098846 | FAM35BP | -4.4 | 0.007831 |
| NM_031958 | KRTAP3-1 | -4.4 | 3.55E-05 |
| NM_001190907 | AKR1D1 | -4.3 | 0.000157 |
| NM_001105069 | ACSM2B | -4.3 | 3.48E-06 |
| NM_177455 | BHLHA15 | -4.3 | 0.000681 |
| NM_003644 | GAS7 | -4.3 | 3.23E-18 |
| NM_004391 | CYP8B1 | -4.3 | 0.006967 |
| NM_001317777 | RIPPLY3 | -4.2 | 2.83E-39 |
| NM_194072 | HILS1 | -4.2 | 1.48E-05 |
| NR_119379 | LOC102723385 | -4.2 | 1.36E-13 |
| NM_003807 | TNFSF14 | -4.2 | 7.12E-07 |
| NR_003682 | MGC70870 | -4.2 | 3.36E-10 |
| NM_017726 | PPP1R14D | -4.2 | 2.26E-61 |
| NM_021243 | ABRACL | -4.2 | 1.60E-07 |
| NM_003167 | SULT2A1 | -4.2 | 5.06E-05 |
| NM_024628 | SLC12A8 | -4.2 | 0.079288 |
| NM_002191 | INHA | -4.2 | 2.71E-08 |
| NM_020957 | PCDHB16 | -4.2 | 0.059896 |
| NM_001017929 | FRMPD2 | -4.1 | 2.79E-13 |
| NM_001013732 | PTCHD4 | -4.1 | 0.027959 |
| NM_001039784 | ADORA2A-AS1 | -4.1 | 1.35E-05 |
| NM_001330148 | GRAP | -4.1 | 0.018415 |
| NR_040073 | MIR181A1HG | -4.1 | 1.03E-08 |
| NM_198455 | SSPO | -4.1 | 0.087487 |
| NM_001010906 | NUGGC | -4.1 | 0.050442 |
| NR_026567 | ESPNP | -4.1 | 0.007432 |
| NM_001166434 | ITIH1 | -4.1 | 0.028493 |
| NM_001079874 | VAV3 | -4.0 | 0.094484 |
| NM_145872 | ASB4 | -4.0 | 2.73E-06 |
| NM_030812 | ACTL8 | -4.0 | 4.56E-05 |
| NM_014344 | FJX1 | -4.0 | 0.016333 |
| NM_001303460 | FGF11 | -4.0 | 1.19E-32 |
| NM_001013742 | DGKK | -4.0 | 0.001757 |
| NM_001328630 | SLC6A9 | -4.0 | 0.00485 |
| NR_027022 | LINC00628 | -4.0 | 0.009867 |
| NM_001287008 | SUSD3 | -4.0 | 0.094312 |
| NM_173846 | TPPP2 | -4.0 | 0.097617 |
| NM_003399 | XPNPEP2 | -4.0 | 0.094484 |
| NR_046094 | LINC01146 | -4.0 | 0.001477 |
| NM_002053 | GBP1 | -4.0 | 0.014905 |
| NM_001166107 | HMGCS2 | -4.0 | 9.39E-12 |
| NR_110826 | KCCAT211 | -4.0 | 0.001277 |
| NM_182758 | WDR72 | -4.0 | 0.094484 |

Supplementary Table 2: Top 100 significant upregulated genes in JQ1 treated HepG2 cells

| **Gene Accession_ID** | **Gene Symbol** | **log_2_FoldChange** | **padj** |
| --- | --- | --- | --- |
| NM_001082483 | EFR3B | 7.0 | 0.030433 |
| NM_213998 | ITGA2B | 6.8 | 0.000124 |
| NR_036494 | LOC643201 | 6.3 | 0.129615 |
| NM_001387 | DPYSL3 | 5.5 | 0.078835 |
| NR_106967 | MIR6077 | 4.8 | 0.000122 |
| NR_125387 | LOC100129636 | 4.6 | 0.001221 |
| NM_001171311 | MAP1A | 4.5 | 2.38E-51 |
| NR_026956 | LINC00663 | 4.5 | 0.084073 |
| NM_178731 | LRRTM4 | 4.4 | 0.016189 |
| NM_001127725 | SEC14L5 | 4.3 | 0.038123 |
| NM_207086 | ADHFE1 | 4.3 | 0.013495 |
| NR_027035 | CEP83-AS1 | 4.3 | 0.046729 |
| NM_001111142 | LRRC73 | 4.3 | 3.12E-31 |
| NM_001206220 | SIPA1L2 | 4.2 | 2.33E-90 |
| NM_005018 | PDCD1 | 4.2 | 0.119476 |
| NM_012616 | OMP | 4.2 | 0.054286 |
| NR_028532 | SNORD71 | 4.2 | 0.023405 |
| NR_034027 | CSTF3-AS1 | 4.2 | 0.051291 |
| RNU1-13P | RNU1-13P | 4.2 | 9.89E-06 |
| NM_001011488 | C10orf54 | 4.1 | 0.037185 |
| NM_027724 | CAGE1 | 4.1 | 0.013257 |
| NR_125415 | UBR5-AS1 | 4.0 | 3.41E-09 |
| NM_001703 | ADGRB2 | 4.0 | 9.44E-06 |
| NM_001075958 | INA | 4.0 | 0.00151 |
| NR_121609 | LOC100507291 | 4.0 | 0.110365 |
| NR_110882 | LOC102724596 | 4.0 | 0.012133 |
| NM_001081330 | DNAH2 | 4.0 | 0.061811 |
| NM_001178166 | CCR4 | 4.0 | 0.048511 |
| NM_001246683 | FOS | 3.9 | 0.092216 |
| NR_015379 | UCA1 | 3.9 | 0.114377 |
| NR_104624 | LOC101929181 | 3.9 | 0.040283 |
| NR_036549 | CCNT2-AS1 | 3.8 | 0.047397 |
| NM_025874 | PLIN5 | 3.8 | 0.003679 |
| NM_001318423 | KLF2 | 3.8 | 8.82E-20 |
| NR_103777 | LOC100996251 | 3.8 | 0.102531 |
| NM_001297766 | KIAA0513 | 3.8 | 4.47E-06 |
| NM_001101098 | SLC22A1 | 3.7 | 3.41E-06 |
| NM_183376 | ARRDC4 | 3.7 | 0.020971 |
| NM_001277325 | NPIPA5 | 3.6 | 5.55E-05 |
| NR_049810 | MIR5087 | 3.6 | 5.11E-45 |
| NM_001039735 | ART5 | 3.6 | 0.026272 |
| NM_144975 | SLFN5 | 3.5 | 0.002372 |
| NM_001033343 | SEC31B | 3.5 | 0.02585 |
| NR_103548 | LUCAT1 | 3.5 | 0.066022 |
| NR_038873 | LOC286059 | 3.5 | 0.048572 |
| NR_003513 | NEAT1 | 3.5 | 0.11806 |
| NM_001039766 | LINC00893 | 3.4 | 0.08564 |
| NM_031476 | CRISPLD2 | 3.4 | 0.051334 |
| NM_001011928 | DPEP2 | 3.4 | 0.00289 |
| NR_034118 | NDUFA6-AS1 | 3.3 | 0.088704 |
| NR_034172 | KANSL1-AS1 | 3.3 | NA |
| NM_153017 | FLJ30679 | 3.3 | 0.092216 |
| NR_130929 | LOC644285 | 3.3 | 0.020486 |
| NM_001128023 | ACSBG1 | 3.2 | 2.87E-05 |
| NM_001271672 | TXNIP | 3.2 | 8.87E-06 |
| NR_015433 | LOC93622 | 3.2 | 3.55E-95 |
| NM_001031693 | HHLA3 | 3.2 | 0.100031 |
| NR_104081 | RNVU1-3 | 3.2 | 0.072379 |
| NM_204948 | LFNG | 3.2 | 0.077223 |
| NM_012551 | EGR1 | 3.1 | 0.090783 |
| NR_110324 | MAP3K14-AS1 | 3.1 | 0.074473 |
| NM_015852 | ZNF117 | 3.1 | 0.075956 |
| NR_104086 | RNVU1-19 | 3.1 | 0.075996 |
| NM_001267718 | IL11 | 3.0 | 0.04415 |
| NR_030737 | TTC3P1 | 3.0 | 1.20E-42 |
| NM_001316309 | KIAA1875 | 2.9 | 0.058852 |
| NM_007559 | BMP8B | 2.9 | 0.025814 |
| NM_001113071 | ZFP36 | 2.9 | 0.000107 |
| NM_001009610 | NUTM2D | 2.9 | 0.042272 |
| NM_001316669 | MAFB | 2.8 | 0.000117 |
| NR_038280 | TCEB3-AS1 | 2.8 | 0.027905 |
| NR_002756 | RNU5A-1 | 2.8 | 0.027087 |
| NM_001301250 | CAPN5 | 2.8 | 0.053136 |
| NM_001199450 | ZC3H6 | 2.8 | 0.003568 |
| NR_002187 | TPI1P2 | 2.8 | 0.115234 |
| NR_046220 | KLHL7-AS1 | 2.7 | 0.083014 |
| NR_110707 | LINC01534 | 2.7 | 0.028797 |
| NM_145466 | GGACT | 2.7 | 0.087601 |
| NR_110001 | LOC101926935 | 2.6 | 0.113729 |
| NM_001184922 | YPEL1 | 2.6 | 0.111344 |
| NM_001320373 | VWA5B2 | 2.6 | 0.102995 |
| NM_001281882 | MT2A | 2.6 | 0.093715 |
| NM_001109151 | KCTD21 | 2.6 | 0.05903 |
| NR_002755 | RNU5D-1 | 2.6 | 0.010785 |
| NM_001039581 | ABCA3 | 2.6 | 1.98E-14 |
| NR_015406 | LINC00654 | 2.6 | 0.027429 |
| NM_001339332 | ABCA5 | 2.5 | 1.43E-06 |
| NM_001017740 | RFESD | 2.5 | 2.59E-05 |
| NM_004155 | SERPINB9 | 2.5 | 0.097003 |
| NM_001279803 | CCDC92 | 2.5 | 0.01424 |
| NM_001097993 | ZC3H10 | 2.5 | 0.065487 |
| NR_036507 | NDUFAF4P1 | 2.5 | 0.11142 |
| NR_028327 | LOC100133331 | 2.5 | 0 |
| NM_182634 | DPY19L2P2 | 2.5 | 0.064864 |
| NM_001267786 | GNG3 | 2.5 | 4.76E-09 |
| NM_138466 | ZNF837 | 2.5 | 0.001088 |
| NM_015675 | GADD45B | 2.5 | 0.086926 |
| NM_001145129 | ZNF192P1 | 2.5 | 0.124449 |
| NR_002830 | GEMIN8P4 | 2.5 | 0.095602 |
| NM_001006582 | TMEM175 | 2.5 | 9.33E-26 |

Supplementary Table 3: Top 100 significant downregulated genes in OTX-015 treated HepG2 cells

| **Gene Accession_ID** | **Gene Symbol** | **log_2_FoldChange** | **padj** |
| --- | --- | --- | --- |
| NM_001002574 | NR1H4 | -6.7 | 0.022287 |
| NM_057116 | PPP2R2C | -6.6 | 0.079325 |
| NM_001107353 | SLC17A2 | -6.3 | 0.027468 |
| NM_003695 | LY6D | -6.2 | 7.56E-06 |
| NM_001192864 | ASB4 | -5.9 | 0.001417 |
| NR_110854 | LOC101927919 | -5.8 | 0.040293 |
| NM_001140039 | FYB | -5.7 | 0.000838 |
| NM_012883 | SULT1E1 | -5.6 | 0.093668 |
| NM_178129 | P2RY8 | -5.5 | 1.15E-07 |
| NM_001318425 | C9orf152 | -5.4 | 0.000937 |
| NM_213736 | PDE3A | -5.4 | 0.065444 |
| NM_001329308 | CLDN2 | -5.4 | 7.62E-05 |
| NM_001008440 | LCP1 | -5.3 | 0.036195 |
| NM_001291153 | XAF1 | -5.3 | 0.094153 |
| NM_032897 | FAM222A-AS1 | -5.2 | 0.017425 |
| NM_001102355 | AGXT | -5.2 | 4.66E-05 |
| NM_018488 | TBX4 | -5.1 | 0.093513 |
| NM_001013010 | PCDHB16 | -5.1 | 0.004027 |
| NM_001258192 | PCYT1B | -5.1 | 0.026344 |
| NM_012334 | MYO10 | -5.1 | 0.000178 |
| NM_001305965 | TRIM22 | -5.1 | 0.037791 |
| NM_001006351 | SSPO | -5.1 | 0.109917 |
| NM_001143947 | UNC93A | -5.0 | 0.035106 |
| NM_001192257 | MBOAT4 | -5.0 | 1.07E-05 |
| NR_125365 | LOC401585 | -4.9 | 0.019021 |
| NR_108049 | CCAT1 | -4.8 | 1.38E-17 |
| NM_005435 | ARHGEF5 | -4.8 | 0.000992 |
| NM_001252337 | FXYD2 | -4.7 | 2.40E-59 |
| NM_000799 | EPO | -4.7 | 0.014848 |
| NM_001048131 | OAS1 | -4.6 | 0.065478 |
| NM_020505 | VAV3 | -4.6 | 0.094153 |
| NM_001242955 | TSPAN32 | -4.6 | 0.097858 |
| NM_024861 | C2orf54 | -4.6 | 0.000565 |
| NM_145364 | AKR1D1 | -4.6 | 4.14E-05 |
| NM_001319603 | SULT2A1 | -4.6 | 0.093668 |
| NM_001257642 | NMI | -4.6 | 0.061601 |
| NM_001101763 | PALM3 | -4.5 | 0.021505 |
| NM_174709 | UPK3A | -4.5 | 0.00752 |
| NM_023580 | EPHA1 | -4.5 | 0.020802 |
| NM_001161442 | SH2D2A | -4.5 | 0.00592 |
| NM_029121 | ATP6V1E2 | -4.5 | 0.001566 |
| NM_001193336 | SEC14L6 | -4.5 | 0.073182 |
| NM_001297615 | UGT2B4 | -4.5 | 0.097858 |
| NM_205191 | CRYBB3 | -4.4 | 0.004804 |
| NM_175444 | FCGBP | -4.4 | 2.92E-26 |
| NM_001140352 | CBLN3 | -4.4 | 0.006036 |
| NM_001278144 | ALOX5AP | -4.4 | 0.000325 |
| NM_001033615 | CTSS | -4.4 | 0.005587 |
| NM_023511 | KRTAP3-1 | -4.4 | 0.036936 |
| NM_001285574 | TLR4 | -4.4 | 0.00063 |
| NM_144748 | ACSM2A | -4.4 | 1.67E-06 |
| NM_001077850 | BCAS1 | -4.3 | 0.000489 |
| NM_001082622 | CYP8B1 | -4.3 | 0.001882 |
| NM_001078863 | DRC7 | -4.3 | 3.79E-11 |
| NM_001185152 | NFE2 | -4.3 | 0.059559 |
| NM_133229 | RIPPLY3 | -4.3 | 0.027268 |
| NM_174357 | IL1RN | -4.2 | 0.041422 |
| NM_133351 | PRSS8 | -4.2 | 0.025565 |
| NR_119379 | LOC102723385 | -4.2 | 0.016491 |
| NM_001046285 | C8A | -4.2 | 0.003017 |
| NR_003682 | MGC70870 | -4.2 | 0.059737 |
| NM_001280009 | AMHR2 | -4.2 | 0.000114 |
| NM_001173084 | GNAT1 | -4.2 | 0.000924 |
| NM_134251 | SLC12A8 | -4.2 | 2.31E-09 |
| NM_001141497 | MBL2 | -4.2 | 0.050318 |
| NM_001013732 | PTCHD4 | -4.2 | 2.00E-34 |
| NM_001140075 | POU2AF1 | -4.2 | 1.07E-24 |
| NM_010544 | IHH | -4.2 | 1.30E-13 |
| NM_001081272 | LDLRAD1 | -4.2 | 0.049504 |
| NM_001017929 | FRMPD2 | -4.2 | 4.14E-10 |
| NM_001178543 | MSI1 | -4.1 | 0.053764 |
| NM_001109266 | FAM229A | -4.1 | 0.000583 |
| NM_174253 | C4BPB | -4.1 | 0.002326 |
| NM_001258059 | GRAP | -4.1 | 0.030047 |
| NM_015660 | GIMAP2 | -4.1 | 0.001191 |
| NM_001044274 | ODAM | -4.1 | 5.57E-19 |
| NM_031479 | INHBE | -4.1 | 1.40E-13 |
| NM_031509 | GSTA1 | -4.1 | 0.029539 |
| NM_022783 | DEPTOR | -4.1 | 0.002389 |
| NM_001305024 | HAVCR1 | -4.1 | 0.029539 |
| NM_001131182 | SLC44A4 | -4.1 | 0.037641 |
| NM_020663 | RHOJ | -4.1 | 5.58E-12 |
| NR_033841 | LOC200772 | -4.1 | 0.017868 |
| NR_120364 | CASC19 | -4.0 | 0.005781 |
| NR_026915 | AADACP1 | -4.0 | 9.16E-26 |
| NM_030812 | ACTL8 | -4.0 | 7.86E-06 |
| NM_201048 | PDIA2 | -4.0 | 0.004656 |
| NM_001192939 | FGF11 | -4.0 | 0.006326 |
| NM_001046006 | SLC22A9 | -4.0 | 0.005369 |
| NM_001076780 | PKD1L2 | -4.0 | 0.06852 |
| NR_110121 | LOC101928791 | -4.0 | 0.002134 |
| NR_109770 | TONSL-AS1 | -4.0 | 0.097119 |
| NM_028340 | SUSD3 | -4.0 | 0.116436 |
| NM_031544 | AMPD3 | -4.0 | 0.000692 |
| NM_173846 | TPPP2 | -4.0 | 0.097119 |
| NM_201032 | XPNPEP2 | -4.0 | 9.60E-40 |
| NM_001315538 | FOXP2 | -4.0 | 0.02471 |
| NM_001002343 | GBP1 | -4.0 | 0.025951 |
| NR_110826 | KCCAT211 | -4.0 | 0.034671 |
| NM_177914 | DGKK | -4.0 | 0.007869 |

Supplementary Table 4: Top 100 significant upregulated genes in OTX-015 treated HepG2 cells

| **Gene Accession_ID** | **Gene Symbol** | **log_2_FoldChange** | **padj** |
| --- | --- | --- | --- |
| NM_014971 | EFR3B | 6.9 | 2.79E-14 |
| NM_000419 | ITGA2B | 6.6 | 7.18E-06 |
| NR_036494 | LOC643201 | 6.5 | 9.02E-06 |
| NM_001387 | DPYSL3 | 5.5 | 8.91E-06 |
| NM_015623 | TANC2 | 4.9 | 0.008643 |
| NM_014723 | SNPH | 4.9 | 0.008985 |
| NM_005252 | FOS | 4.7 | 5.08E-14 |
| NM_002373 | MAP1A | 4.7 | 0.014841 |
| NM_002775 | HTRA1 | 4.6 | 0.027408 |
| NM_003949 | HAP1 | 4.6 | 0.021505 |
| NM_032727 | INA | 4.6 | 0.021505 |
| NM_015193 | ARC | 4.5 | 0.027571 |
| NM_001703 | ADGRB2 | 4.4 | 0.037445 |
| NR_027035 | CEP83-AS1 | 4.3 | 0.013061 |
| NR_106967 | MIR6077 | 4.3 | 0.046901 |
| NM_144650 | ADHFE1 | 4.2 | 0.050687 |
| NR_040100 | ENTPD3-AS1 | 4.2 | 0.052927 |
| NM_022165 | LIN7B | 4.1 | 0.073373 |
| NM_201589 | MAFA | 4.1 | 0.064822 |
| NM_003378 | VGF | 4.1 | 0.077668 |
| NR_049810 | MIR5087 | 4.0 | 0.02755 |
| NR_036549 | CCNT2-AS1 | 4.0 | 0.000482 |
| NR_026703 | VTRNA1-1 | 3.9 | 0.093513 |
| NM_001040167 | LFNG | 3.9 | 1.13E-07 |
| NM_001320173 | C14orf37 | 3.8 | 0.05297 |
| NM_001324159 | DPEP2 | 3.8 | 0.048651 |
| NM_004466 | GPC5 | 3.8 | NA |
| NR_120539 | LOC101928008 | 3.8 | NA |
| NM_014604 | TAX1BP3 | 3.8 | NA |
| NR_110841 | LOC101927730 | 3.8 | NA |
| NM_001001710 | FAM166A | 3.8 | 0.067636 |
| NR_121645 | RNVU1-8 | 3.7 | 0.01715 |
| NM_001267718 | IL11 | 3.6 | 0.020788 |
| NM_183376 | ARRDC4 | 3.6 | 1.77E-18 |
| NR_103548 | LUCAT1 | 3.6 | 0.027174 |
| NM_014692 | SEC14L5 | 3.5 | 0.094799 |
| NM_001964 | EGR1 | 3.5 | 1.13E-70 |
| NM_001286688 | ABLIM2 | 3.5 | 0.09039 |
| NM_001297766 | KIAA0513 | 3.5 | 7.34E-05 |
| NM_005167 | PPM1J | 3.5 | 0.098474 |
| NR_125415 | UBR5-AS1 | 3.5 | 0.098474 |
| RNU1-13P | RNU1-13P | 3.5 | 0.030756 |
| NR_039795 | MIR4651 | 3.4 | 0.112735 |
| NM_001720 | BMP8B | 3.4 | 0.03164 |
| NM_003407 | ZFP36 | 3.4 | 1.48E-07 |
| NR_038873 | LOC286059 | 3.4 | 0.001786 |
| NM_001009565 | CDKL4 | 3.4 | 0.104489 |
| NM_004093 | EFNB2 | 3.4 | 0.107991 |
| NM_145051 | RNF183 | 3.4 | 0.112615 |
| NM_030907 | RSG1 | 3.4 | 0.124105 |
| NR_038379 | LOC554206 | 3.4 | 0.12511 |
| NR_130929 | LOC644285 | 3.4 | 0.12511 |
| NR_002802 | NEAT1 | 3.3 | 0 |
| NM_005461 | MAFB | 3.3 | 0.01214 |
| NM_001039766 | LINC00893 | 3.3 | 0.129588 |
| NM_001013706 | PLIN5 | 3.3 | 0.003182 |
| NR_002756 | RNU5A-1 | 3.3 | 0.000254 |
| NM_001009610 | NUTM2D | 3.2 | 0.015507 |
| NR_130910 | YPEL1 | 3.2 | 0.002187 |
| NR_015433 | LOC93622 | 3.1 | 8.19E-29 |
| NM_020731 | AHRR | 3.1 | 0.069 |
| NM_001316309 | KIAA1875 | 3.1 | 0.085265 |
| NM_015162 | ACSBG1 | 3.1 | 0.038139 |
| NM_002965 | S100A9 | 3.0 | 0.084414 |
| NM_001031693 | HHLA3 | 3.0 | 0.031078 |
| NM_001313972 | TXNIP | 3.0 | 4.02E-80 |
| NM_001286115 | THEMIS2 | 3.0 | 0.014216 |
| NR_104081 | RNVU1-3 | 2.9 | 0.126302 |
| NM_015852 | ZNF117 | 2.8 | 6.75E-37 |
| NM_006176 | NRGN | 2.8 | 0.004462 |
| NM_145245 | EVI5L | 2.8 | 9.17E-08 |
| NR_104086 | RNVU1-19 | 2.8 | 0.000816 |
| NM_198581 | ZC3H6 | 2.7 | 1.13E-05 |
| NR_002755 | RNU5D-1 | 2.7 | 0.000765 |
| NR_104076 | RNVU1-15 | 2.7 | 1.79E-05 |
| NR_110199 | DARS-AS1 | 2.7 | 0.087688 |
| NM_015675 | GADD45B | 2.7 | 1.09E-05 |
| NM_020928 | ZSWIM6 | 2.7 | 1.25E-21 |
| NM_001324112 | CYP7B1 | 2.7 | 0.0747 |
| NM_001142854 | SPATC1L | 2.6 | 0.088129 |
| NM_017731 | OSBPL7 | 2.6 | 0.022009 |
| NR_034172 | KANSL1-AS1 | 2.6 | 0.081559 |
| NR_002830 | GEMIN8P4 | 2.6 | 0.098465 |
| NR_038334 | LOC100505715 | 2.6 | 0.102161 |
| NM_004155 | SERPINB9 | 2.6 | 2.21E-42 |
| NM_182634 | DPY19L2P2 | 2.6 | 0.096404 |
| NM_001303125 | ZC3H10 | 2.6 | 4.04E-10 |
| NM_001029859 | KCTD21 | 2.6 | 2.91E-06 |
| NM_080725 | SRXN1 | 2.5 | 7.13E-193 |
| NR_036507 | NDUFAF4P1 | 2.5 | 0.106046 |
| NM_147162 | IL11RA | 2.5 | 0.001383 |
| NM_052878 | IZUMO4 | 2.5 | 0.035474 |
| NM_013376 | SERTAD1 | 2.5 | 1.34E-10 |
| NM_001004431 | METRNL | 2.5 | 0.041019 |
| NM_001031737 | CCDC78 | 2.5 | 0.126567 |
| NM_032701 | SUV420H2 | 2.4 | 0.000596 |
| NM_174891 | C14orf79 | 2.4 | 0.082735 |
| NM_001330358 | MTHFR | 2.4 | 2.79E-21 |
| NM_182980 | OSGIN1 | 2.4 | 3.49E-39 |
| NR_028327 | LOC100133331 | 2.4 | 0.126399 |

**Supplementary methods**

**Transwell assay**

Transwell assay was conducted by 24-well Transwell inserts (Corning, NY, USA; 351152) in transfected Huh7 cells. After siRNA transfected for 48 hours, cells were stained with calcein AM. The cells were seeded into the upper chamber that 8 μm pore size inserts, and allowed to migrate to the bottom chamber containing 10% FBS. After incubation for 24 h, the migrated cells attached to the lower membrane surface were photographed. The data represent three independent experiments (n=3). **, *p* < 0.01.
